# Assessing the Impact of Relapse, Reinfection and Recrudescence on Malaria Eradication Policy: A Bifurcation and Optimal Control Analysis

**DOI:** 10.3390/tropicalmed7100263

**Published:** 2022-09-24

**Authors:** Hengki Tasman, Dipo Aldila, Putri A. Dumbela, Meksianis Z. Ndii, Fatmawati, Faishal F. Herdicho, Chidozie W. Chukwu

**Affiliations:** 1 Department of Mathematics, Universitas Indonesia, Depok 16424, Indonesia; 2 Department of Mathematics, University of Nusa Cendana, Kupang-NTT 85361, Indonesia; 3 Department of Mathematics, Faculty of Science and Technology, Universitas Airlangga, Jakarta 12920, Indonesia; 4 Department of Mathematics, Wake Forest University, Winston-Salem, NC 27109, USA

**Keywords:** malaria, relapse, reinfection, recrudescence, backward bifurcation, hysteresis, optimal control, cost-effectiveness

## Abstract

In the present study, we propose and analyze an epidemic mathematical model for malaria dynamics, considering multiple recurrent phenomena: relapse, reinfection, and recrudescence. A limitation in hospital bed capacity, which can affect the treatment rate, is modeled using a saturated treatment function. The qualitative behavior of the model, covering the existence and stability criteria of the endemic equilibrium, is investigated rigorously. The concept of the basic reproduction number of the proposed model is obtained using the concept of the next-generation matrix. We find that the malaria-free equilibrium point is locally asymptotically stable if the basic reproduction number is less than one and unstable if it is larger than one. Our observation on the malaria-endemic equilibrium of the proposed model shows possible multiple endemic equilibria when the basic reproduction number is larger or smaller than one. Hence, we conclude that a condition of a basic reproduction number less than one is not sufficient to guarantee the extinction of malaria from the population. To test our model in a real-life situation, we fit our model parameters using the monthly incidence data from districts in Central Sumba, Indonesia called Wee Luri, which were collected from the Wee Luri Health Center. Using the first twenty months’ data from Wee Luri district, we show that our model can fit the data with a confidence interval of 95%. Both analytical and numerical experiments show that a limitation in hospital bed capacity and reinfection can trigger a more substantial possibility of the appearance of backward bifurcation. On the other hand, we find that an increase in relapse can reduce the chance of the appearance of backward bifurcation. A non-trivial result appears in that a higher probability of recrudescence (treatment failure) does not always result in the appearance of backward bifurcation. From the global sensitivity analysis using a combination of Latin hypercube sampling and partial rank correlation coefficient, we found that the initial infection rate in humans and the mosquito infection rate are the most influential parameters in determining the increase in total new human infections. We expand our model as an optimal control problem by including three types of malaria interventions, namely the use of bed net, hospitalization, and fumigation as a time-dependent variable. Using the Pontryagin maximum principle, we characterize our optimal control problem. Results from our cost-effectiveness analysis suggest that hospitalization only is the most cost-effective strategy required to control malaria disease.

## 1. Introduction

In many parts of the world, diseases spread by intermediary vectors such as mosquitoes, are still a serious problem for policymakers. Some examples of mosquito-borne diseases include yellow fever, dengue, zika, malaria, filariasis, etc. [1]. Malaria is an example of a vector-borne disease caused by a mosquito (female anopheles mosquito). It is still a problem in many countries, where most cases are found in Africa (almost 95% of cases). The rest are spread out in parts of Latin America and Southeast Asia, including Indonesia [2]. Malaria causes a global problem that threatens hundreds of millions of people every year, mainly children under five years old, pregnant women, travelers who go to malaria-endemic areas without protection, and patients with other acute conditions (HIV, diabetes, etc.). Four types of *Plasmodium* cause malaria (*P. vivax, P. falciparum, P. malaria, P. ovale* [3,4]. Malaria involves both humans and mosquitoes in their life cycle. The term sporogonic cycle refers to the life cycle in the mosquito’s body. Furthermore, the life cycle in the liver and human blood are called the exo-erythrocytic cycle and the erythrocytic cycle, respectively. Plasmodium will destroy the liver and red blood cells when it gets into the bloodstream and causes symptoms such as fever and flu-like illness, muscle aches, headache, tiredness, and even death.

Stakeholders have introduced many different types of efforts in many countries to control the spread of malaria, focusing on preventing mosquitoes’ bites and controlling the population of mosquitoes. These interventions include using insecticide-treated bed nets (ITNs), antimalarial drugs, or fumigation, which is already banned in several areas because it will harm the non-targeted population. Recent studies proposed transmission-blocking interventions (TBIs) to stop the transmission of gametocytes from humans to mosquitoes [5]. The high incidence of malaria worldwide every year suggests that these types of intervention are still not producing optimal results, so continuous development is needed. The difficulties of malaria control are mainly due to mosquitoes’ good adaptability to environmental changes (for example, the ability to build resistance to insecticides) [6,7]. Another possible reason is the different characteristics of each *Plasmodium* make it difficult for antimalarial drugs to be effective for everyone.

Another thing to be considered in malaria reduction strategy besides the lack of hospital capacity [8] is the recurrent phenomena that frequently appear in the infected human. These recurrent phenomena (relapse, reinfection, and recrudescence) develop another complex problem in malaria eradication policy [9], which makes malaria harder to eradicate. Authors in [10] state that 21.5% of infected individual by *P. falciparum* in Myanmar experience recurrence 63 days after treatment, while for *P. vivax*, the recurrence rate is 31.5%. Relapse occurs due to the manifestation of the emergence of malaria infection as a result of reactivation of the *Plasmodium* parasite in the human liver. Relapse occurs mostly in malaria infection with *P. vivax* and *P. ovale*. Reinfection occurs from a new bite of a female *anopheles* mosquito to an infected individual. On the other hand, recrudescence is defined as the reactivation of malaria due to incomplete eradication of *Plasmodium* after treatment. Although genetic reasoning of the cause of recurrent malaria has not yet been well defined, proper malaria-monitoring treatment should be implemented to prevent any recurrent episodes in infected individuals [11].

Since the model by Ross [12] and Macdonald [13], many authors have developed more complex mathematical models to understand the mechanism of malaria transmission. For example, Tumwiine et al. [14] introduced a host–vector model for malaria transmission, which considers temporary immunity. An analysis regarding the existence and stability of the equilibrium points was conducted by Tumwiine et al. They found that malaria will always exist if the basic reproduction number is larger than one. Traore et al. [15] considered a structured mosquito population and variation in the temperatures in their model. They found that the global behavior (persistence of malaria) depends on two thresholds: the reproduction of mosquitoes and the basic reproduction number. Aldila et al. [4] considered vector-bias phenomena in their model and used an optimal control approach to find the best possible strategies to eradicate malaria. Several other mathematical models for malaria have also introduced the consideration of several factors, such as human mobility [16], control strategies [17], temperature [18], vector-bias [19], superinfection [20], seasonal factors [21,22], impact of vaccines and transmission-blocking drugs [23], social hierarchy [24], etc.

Although there are many mathematical models for malaria transmission, not much research considers recurrent phenomena in their model. Niger and Gumel [25] considered reinfection in their model and found that reinfection can trigger a backward bifurcation phenomenon. Chamcod and Beier [26] considered relapses, treatment, seasonality, and G6PD in their model. Their results indicate that increasing the deficiency of G6PD will increase the number of infected humans. Ghosh et al. [27] considered two types of recurrent on their proposed model, namely relapse and reinfection. Cost-effectiveness analysis was considered in their model, to find the most effective strategies for the malaria eradication program. Woldegerima et al. [5] considered relapse in their model with transmission-blocking drug (TBD) intervention. They used incidence data from Sub-Saharan Africa to validate their model and found that TBD combined with other interventions could suppress the spread of malaria in a few years. Recently, Wang et al. [28] introduced an age-structure malaria model considering vaccination and contact prevention as eradication efforts. To date, not many mathematical models include three types of infection in malaria: recrudescence, relapse, and reinfection in a single model. Recently, [29] introduced their malaria model involving the three types of infection mentioned before. A mathematical analysis regarding the equilibrium points, the basic reproduction number, the backward bifurcation phenomena, and the global stability of the endemic equilibrium was discussed in detail. However, the authors in [29] did not yet include limited hospital capacity in their model. This factor is essential since health services will be able to be provided more optimally if the hospital capacity is also adequate in number.

Motivated by the above discussions, this paper aims to fill the gap in the mentioned references, where we consider all recurrent phenomena (relapse, reinfection, and recrudescence) together in one model. Limitation of hospital capacity to treat infected individuals is accommodated into our model as a saturated hospitalization function. We analyze the existence and stability criteria of our equilibrium points thoroughly. Bifurcation analysis on our model shows three possible types of bifurcation phenomena at basic reproduction numbers equal to one, namely forward bifurcation, backward bifurcation, and forward bifurcation with hysteresis. To calibrate our mathematical model, we estimated our parameter using malaria incidence data from the Wee Luri Health Center in Indonesia. In addition, we consider our second model, namely the malaria intervention model, as an optimal control problem. Cost-effectiveness analyses were computed to determine the most cost-effective strategy necessary to control malaria leading to its eradication. The paper is organized as follows: Formulation of the malaria model as a system of ordinary differential equations is given in Section 2 along with its fundamental properties. In Section 3, we analyze the existence criteria of our model and how it relates to the basic reproduction number. We also conduct parameter estimation on the infection parameters in Section 3. Bifurcation analysis using Castillo–Song theorem [30] is conducted analytically in Section 4 and supported with some numerical experiments to give a visualization of our results. Optimal control characterization of our intervention model is given in Section 6 and followed with optimal control simulations. We wrap up our work with discussion and conclusion in Section 8.

## 2. Mathematical Model Formulation and Parameter Estimation

### 2.1. The Mathematical Model

To develop a mathematical model which describes the transmission process of malaria considering relapse, reinfection, and recrudescence, we divided our human population based on their health status into seven classes of the human population, namely the susceptible class of human at time *t*, denoted by S(t); the exposed class of human at time *t*, denoted by E(t); the dormant and latent classes of humans at time *t*, denoted by D(t) and L(t), respectively; the infected class at time *t*, denoted by I(t); the treated class of human at time *t*, denoted by T(t); and the recovered class of human, denoted by R(t). Hence, the total of the human population at time *t*, denoted by N(t), is given by

(1) N(t)=S(t)+E(t)+D(t)+L(t)+I(t)+T(t)+R(t).

In addition, the mosquito population is divided into susceptible and infected classes of mosquitoes, which is denoted by U(t) and V(t), respectively. Note that we do not consider a recovered class for the mosquito population due to the short life expectancy of female anopheles mosquitoes, which is only ±10−14 days [31]. Hence, the total mosquito population (denoted by M(t)) is given by

(2) M(t)=U(t)+V(t).

**Figure 1:**
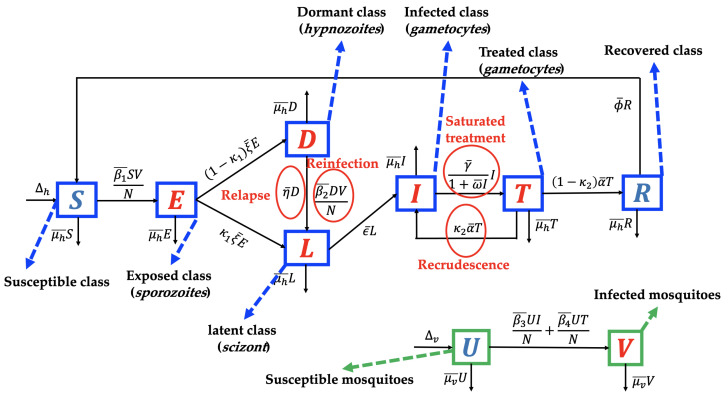
Transmission diagram of malaria disease considering relapse, reinfection, recrudescence, and saturated treatment.

We use the transmission diagram shown in Figure 1 to construct our model. The derivation of the model is given in detail as follows.

The individuals of the susceptible human class are assumed to be recruited at a constant number $\Delta_{h}$. We assume that all recruitment rates of humans from newborns are entering the susceptible class only. However, several reports [32,33] indicate a possibility of mother-to-children vertical transmission. We assume that no migration is involved in our model, both in the human and mosquito populations. In addition, the susceptible population may decrease due to acquiring malaria through direct contact with an infected mosquito *V* with a successful contact rate of ${\bar{\beta }}_{1}$. We consider the standard incidence rate for the interaction between humans and mosquitoes in the form of $\frac{{\bar{\beta }}_{1}SV}{N}$. These individuals are then transferred to the exposed class. In the exposed class, *Plasmodium* is in the *sporozoites* form. Hence, individuals in *E* are not yet capable of transmitting malaria to the susceptible mosquito. We assume that the intrinsic incubation period of *E* is denoted by ${\bar{\xi }}^{-1}$ and takes about 7–30 days [34]. In our model, it is possible that *sporozoites* in *E* may transformed into different type of *Plasmodium*, namely *hypnozoites* (transferred into *D* with a proportion of $\kappa_{1}$) and *scizont* (transferred into *L* with a proportion of $1-\kappa_{1}$).

*Plasmodium* in the form of *sporozoites* is assumed to be dormant in the liver of individuals in class *D*. The dormant period of *Plasmodium* in the human liver varies depending on the type of *Plasmodium* that infects the human (*P. vivax* or *P. ovale*). In our model, we assume that exposed individuals who do not experience a dormant stage are given in a proportion of $\kappa_{1}$ and transferred to *L*, while a $1-\kappa_{1}$ proportion of *E* that experience the dormant are stage transferred to *D*. As we mentioned earlier, relapse occurs due to reactivation of *Plasmodium* in the liver. Hence, after a dormancy period of ${\bar{\eta }}^{1}$, individuals in *D* go to *L* due to relapse phenomena. In addition, infected individuals in the dormant class can also experience reinfection with a transmission rate of ${\bar{\beta }}_{2}$ due to direct contact with the infected mosquito. Hence, the total number of recruitment on latent class *L* is given by $\kappa_{1}\bar{\xi }E+\bar{\eta }D+\bar{\beta_{2}}DV/N$.

After passing through the schizont phase, Plasmodium will develop to attack the human body and enter the gametocyte phase. In this phase, humans will begin to show symptoms of malaria such as fever and flu-like illness, muscle aches, tiredness, depending on each individual’s response to malaria. In our model, groups with characteristics such as this are in the infected group (I). The number of individuals in *I* increases due to transition from the latent group at a rate of $\bar{\epsilon }$. Our model assumes that a portion of *I* will receive hospitalized care to get better treatment. The hospitalization action gives rise to a new problem in many countries that have a low hospital bed capacity ratio. Hospital bed capacity ratio is defined as the average number of beds available in hospitals per 1000 population in a country [35]. For example, Nigeria, as the country with the highest malaria cases in 2019 (27% cases worldwide) [2] had a hospital bed capacity ratio of only 0.5 [35]. This means that there are only five beds available in a hospital for 10,000 people in Nigeria on average. In addition, four other countries with the highest malaria cases in the world in 2020, namely the Democratic Republic of the Congo (12%), Uganda (5%), Mozambique (4%), and Niger (3%) [2], only have hospital bed capacity ratios of 0.8, 0.5, 0.7, and 0.4, respectively [35]. Therefore, when the number of cases increases significantly, the number of people who can be hospitalized will certainly not be optimal. To accommodate the limited hospitalization resources, we use the following assumption: Let the hospitalization intervention as a function depending on the number of infected individuals, f(I). We assume that increasing the number of infected individuals will decrease the hospitalization rate. Hence, we have that $\frac{df(I)}{dI}<0$. In addition, we assume $\lim_{I\to \infty }f\left(I\right)=0$, which describes a situation where hospitalization is impossible to conduct when the number of infected individuals is out of control. Hence, we choose $f\left(I\right)=\frac{\bar{\gamma }}{1+\bar{\omega }I}I$, where $\bar{\gamma }$ and $\bar{\omega }$ present the hospitalization rate and half-saturation parameters, respectively.

The next class is the treated class. This class increased due to hospitalization from *I* individuals and decreased after the treatment period, denoted by ${\bar{\alpha }}^{-1}$. In our model, we assume that after following the treatment program, an individual in *T* will go to the *R* class due to treatment success with a proportion of $1-\kappa_{2}$ and return to *I* due to recrudescence with a proportion of $\kappa_{2}$. As we mentioned before, recrudescence occurs when the number of *Plasmodium* in the treated individual can no longer be detected and then becomes detectable again. Our model assumes that this recrudescence leads to malaria treatment failure. The last class is the recovered class, which increases due to treatment success from *I*. It decreases due to the end of the temporary period of immunity against malaria, whose rate is denoted by $\bar{\phi }$. We assume that all classes of the human population decreased due to the natural death rate, denoted by $\bar{\mu_{h}}$. In addition, we neglect the death rate induced by malaria.

Unlike the human population, the mosquito population is divided into two classes in our model, namely susceptible (U) and infected (V) mosquitoes. We assume that susceptible mosquitoes can get infected by malaria due to biting infected humans *I* and *T*, with rates of $\beta_{3}$ and $\beta_{4}$, respectively. Both of these mosquito classes decreased due to the natural mosquito death rate, denoted by $\bar{\mu_{v}}$. A recovered class in mosquitoes was not considered in our proposed model. This is because the infected female Anopheles mosquito will continue to be infected for the rest of her life [36]. Based on transmission diagram in Figure 1 and using all mentioned assumptions above, the mathematical model for malaria transmission, considering relapse, reinfection, recrudescence, and saturated hospitalization rate is given in Supplementary File (Equation (S1)). Please see Table 1 for a description of the parameters.

### 2.2. Parameter Estimation

In this section, we fitted the model against monthly data of malaria incidence from Wee Luri Health Center, Central Sumba, Indonesia. We use the nonlinear-squared fitting by minimizing the model solutions and the data [45]. In our model, we fit the number of treated individuals (T(t)) using the best fit parameter $\beta_{1}$, $\beta_{2}$, $\beta_{3},\beta_{4}$ and γ. Let $\hat{\Theta }=\left(\theta_{1},\theta_{2},\ldots ,\theta_{5}\right)$ present $\beta_{1}$, $\beta_{2}$, $\beta_{3}$, $\beta_{4}$, and γ, respectively, and be the set of parameters that minimize the sum of squared error between the data $T_{t_{i}}=T_{t_{1}},T_{t_{2}},\ldots ,T_{t_{n}}$ and the model solutions $f(t_{i},\Theta )$ of our malaria model, which is

(3) $$\hat{\Theta }=\mathrm{argmin}\sum_{i=1}^{n}{\left(f\left(t_{i},\Theta \right)-T_{t_{i}}\right)}^{2}.$$

**Figure 2:**
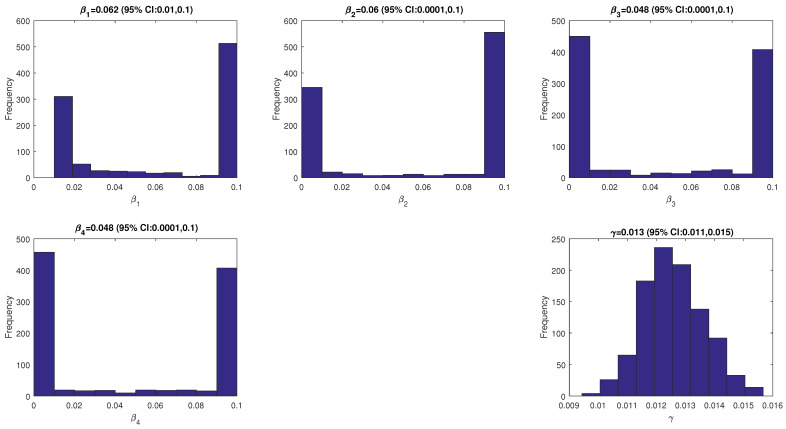
The values of the fitted parameters ($\beta_{1},\beta_{2},\beta_{3},\beta_{4}$, and γ).

**Figure 3:**
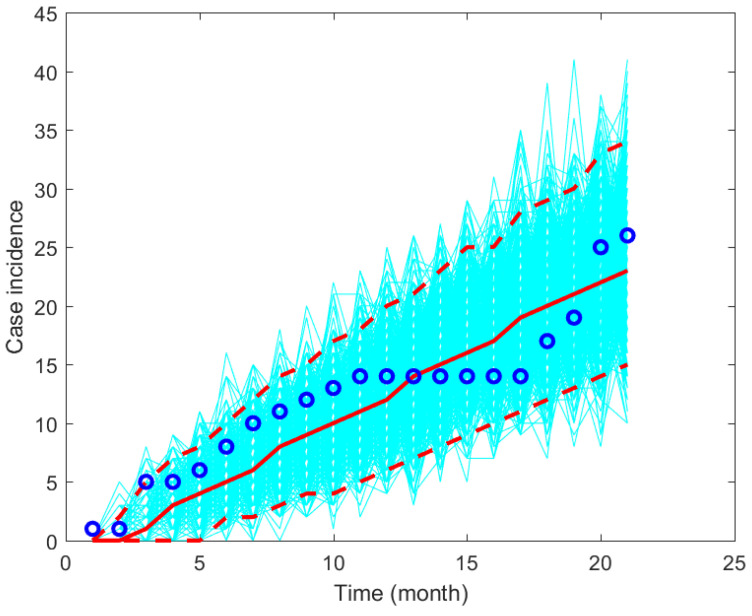
Plot simulation results and the data using the fitted parameter values based on monthly data from Wee Luri Health Center, Sumba, Indonesia.

The model’s solution is obtained by solving the model using the ode45 function in MATLAB. The other parameter values are given in Table 1. The fitted parameter values are given in Figure 2 and a plot of the data and model’s solutions is given in Figure 3. It can be seen that the data is still in the range of the confidence intervals given in Figure 3.

## 3. Mathematical Analysis

In this chapter, analysis of the mathematical model begins by non-dimensionalizing the model on both variables and parameters. After the non-dimensionalization process, an analysis of the existence of all equilibrium points is discussed in relation to the basic reproduction number ($R_{0}$). In addition, we analyzed the effects of three types of recurrences on the size of $R_{0}$.

### 3.1. Non-Dimensional Model

To facilitate an easier analytical study, we utilized a non-dimensionalization process of the variables and parameters in the original model (Equation (S1) in Supplementary File). By assuming a constant total population and using some new transformations (see the Supplementary Section S1 for the details), the model can be simplified into less dimensions (please see Equation (S4) in the Supplementary File).

With this non-dimensionalization process, we reduce our system from 9 to 7 variables and from 17 to 14 parameters. Since our non-dimensionalized model presents the number of humans and mosquitoes, then the value should be non-negative. Hence, it is crucial to show that the non-dimensionalized model will always give a non-negative solution for all time t≥0. These properties are stated in the following theorem.

**Theorem** **1.** *With non-negative initial condition s>0,e≥0,d≥0,l≥0,i≥0,m≥0 and v≥0, then any solution of the non–dimensionalized model will always be non-negative for all time t≥0.*

**Proof**. Please see Supplementary Section S1 for the proof. □

Since the total population is always constant, and each variable in the non-dimensionalized malaria model (see Supplementary Section S1, Equation (S4)) always non-negative, then we have that each variable is bounded at $\left[0,1\right]$.

### 3.2. Malaria-Free Equilibrium and the Basic Reproduction Number

In this section, we aim to determine the malaria-free equilibrium point of the non-dimensionalized model, and the respective basic reproduction number $\left(R_{0}\right)$. The malaria-free equilibrium point is given by

(4) $$E_{1}=\left(s^{+},e^{+},d^{+},l^{+},i^{+},m^{+},v^{+}\right)=\left(1,0,0,0,0,0,0\right).$$

Next, we calculate the basic reproduction number, which is defined as the expected number of secondary malaria cases caused by one primary malaria case in a single infection period in a completely susceptible population [4]. The basic reproduction number provides a dimensionless threshold, which becomes the endemic indicator of the respected epidemiological models. In many epidemiological models [46,47,48,49], it was discovered that there is a chance that the disease will disappear when the basic reproduction number is less than one, and always exist when it is larger than one. Hence, it is important to find the $R_{0}$, so we can determine whether malaria will exist or disappear from the population. The basic reproduction number is taken from the spectral radius of the next-generation matrix of the respective model. Using next-generation matrix approach [50,51,52], the $R_{0}$ is given by

(5) $$R_{0}=\frac{\xi \;\left(\kappa_{1}\mu_{h}+\eta \right)\left(\alpha \;\beta_{3}+\gamma \;\beta_{4}+\beta_{3}\mu_{h}\right)\beta_{1}}{\left(\xi +\mu_{h}\right)\left(\eta +\mu_{h}\right)\left(\mu_{h}+1\right)\left(\alpha \;\gamma \;\left(1-\kappa_{2}\right)+\mu_{h}\alpha +\gamma \;\mu_{h}+{\mu_{h}}^{2}\right)\mu_{v}}.$$

It is easy to show that our model satisfies the five conditions described in [50]. Hence, using the result in [50], we understand that whenever the basic reproduction number is less than one, we will have a chance to eliminate malaria from the environment. On the other hand, if the basic reproduction number is larger than one, then we will always have malaria in the environment.

From the form of $R_{0}$ in (5), we can see that the reinfection parameter $\beta_{2}$ does not appear. Hence, we can conclude that reinfection phenomena do not affect the size of the basic reproduction number. In addition, we can see that $R_{0}$ is linearly proportional to all infection parameters, except for $\beta_{2}$$\left(\beta_{1},\beta_{3},\beta_{4}\right)$. To give further analysis on the impact of recurrence phenomena on the malaria transmission on our proposed model, we will analyze the basic reproduction number of a simple case using our proposed model.

**Figure 4:**
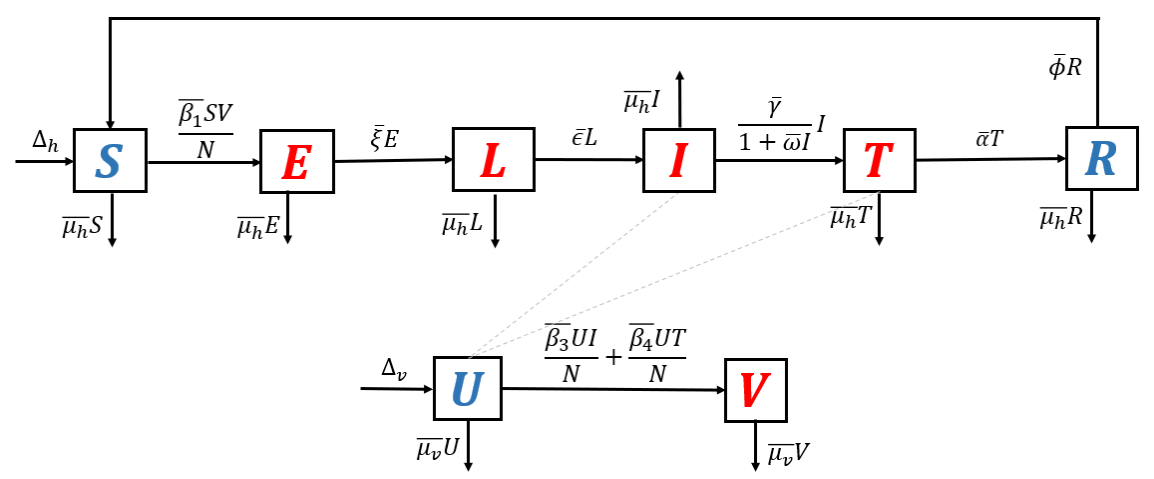
Transmission diagram of malaria disease without any recurrence phenomena.

**Figure 5:**
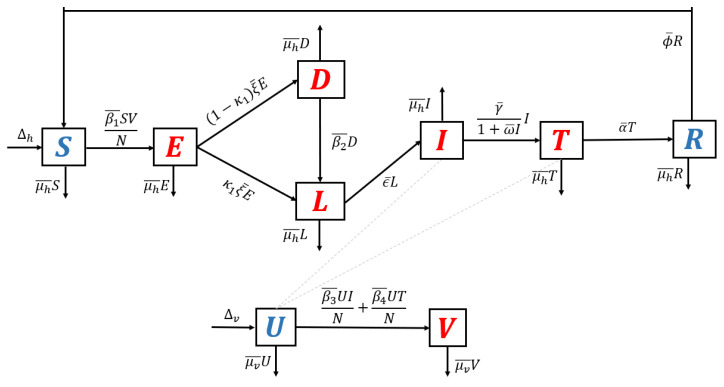
Transmission diagram of malaria disease with reinfection only, without relapse and recrudescence.

**Figure 6:**
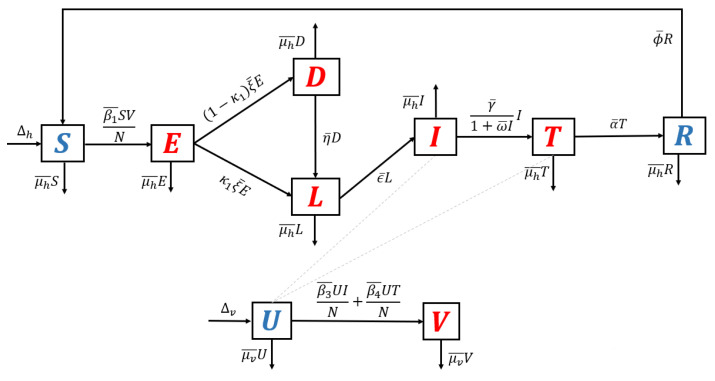
Transmission diagram of malaria disease with relapse only, without reinfection and recrudescence.

**Figure 7:**
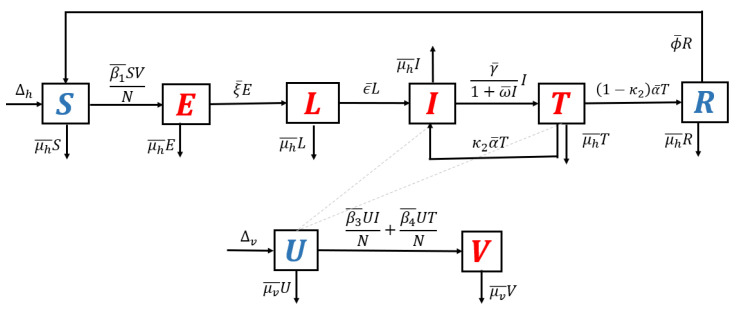
Transmission diagram of malaria disease with recrudescence only, without relapse and reinfection.

1. **Model without recurrence.** When all recurrence phenomena are not involved in the model, i.e., $\bar{\beta_{2}}=0,\;\bar{\eta }=0$, and $\kappa_{2}=0$, then the transmission diagram in Figure 1 is reduced to the transmission diagram in Figure 4.
  Using the parameter transformation in Supplementary Section S1, the basic reproduction number of the malaria model using transmission diagram in Figure 4 is given by
  (6) $$R_{0}^{\mathrm{standard}}=\frac{\xi \;\left(\alpha \;\beta_{3}+\gamma \;\beta_{4}+\beta_{3}\mu_{h}\right)\beta_{1}}{\left(\xi +\mu_{h}\right)\left(\mu_{h}+1\right)\left(\alpha +\mu_{h}\right)\left(\mu_{h}+\gamma \right)\mu_{v}}.$$
  To give further interpretation of $R_{0}^{\mathrm{standard}}$, Equation (6) can be rewritten as follows:
  $$\begin{array}{cc} R_{0}^{\mathrm{standard}}\; & =\underset{\mathrm{infection in human}}{\underset{︸}{\left\{\frac{\beta_{1}}{\mu_{v}}\right\}}}\underset{\mathrm{infection in mosquitoes}}{\underset{︸}{\left\{\frac{\beta_{3}}{\mu_{h}+\gamma }+\frac{\gamma \beta_{4}}{\left(\alpha +\mu_{h}\right)\left(\gamma +\mu_{h}\right)}\right\}}}\;\;\;\underset{\mathrm{life time of}\;e}{\underset{︸}{\left\{\frac{1}{\xi +\mu_{h}}\right\}}}\;\;\;\underset{\mathrm{Iuput}-\mathrm{output ratio in}\;l}{\underset{︸}{\left\{\frac{\xi }{1+\mu_{h}}\right\}}}. \end{array}$$
  It is clear to see that $R_{0}^{\mathrm{standard}}$ is a result of multiplication between the number of new infected humans, new infected mosquitoes, and the lifetime of the exposed and latent classes. It can be seen that the saturated parameter of the treatment term does not appear in $R_{0}^{\mathrm{standard}}$.
2. **Model with reinfection only.** When the malaria model in the transmission diagram in Figure 1 includes reinfection only, without relapse and recrudescence, then the transmission diagram becomes that depicted in Figure 5.
  Calculating the basic reproduction number of the non-dimensional form of the model from the transmission diagram in Figure 5, we have
  (7) $$R_{0}^{\mathrm{reinfection}}=\frac{\xi \;\left(\alpha \;\beta_{3}+\gamma \;\beta_{4}+\beta_{3}\mu_{h}\right)\beta_{1}}{\left(\xi +\mu_{h}\right)\left(\mu_{h}+1\right)\left(\alpha +\mu_{h}\right)\left(\mu_{h}+\gamma \right)\mu_{v}}=R_{0}^{\mathrm{standard}}.$$
  It can be seen that $R_{0}^{\mathrm{reinfection}}=R_{0}^{\mathrm{standard}}$, which means that reinfections do not change the standard basic reproduction number.
3. **Model with relapse only.** With the same approach as before, when reinfection and recrudescence are not involved, $\bar{\beta_{2}}=0$ and $\kappa_{2}=0$. Based on this, the transmission diagram in Figure 1 changes to Figure 6.
  The basic reproduction number of a non-dimensional model based on transmission diagram in Figure 6 is given by
  (8) $$R_{0}^{\mathrm{relapse}}=\frac{\xi \;\left(\kappa_{1}\mu_{h}+\eta \right)\left(\alpha \;\beta_{3}+\gamma \;\beta_{4}+\beta_{3}\mu_{h}\right)\beta_{1}}{\left(\xi +\mu_{h}\right)\left(\eta +\mu_{h}\right)\left(\mu_{h}+1\right)\left(\alpha \;\gamma +\alpha \;\mu_{h}+\gamma \;\mu_{h}+{\mu_{h}}^{2}\right)\mu_{v}}=\underset{\mathrm{effect of relapse}}{\underset{︸}{\left\{\frac{\kappa_{1}\mu_{h}+\eta }{\mu_{h}+\eta }\right\}}}\;\;\;R_{0}^{\mathrm{standard}}.$$
  Since $\frac{\kappa_{1}\mu_{h}+\eta }{\mu_{h}+\eta }<1$, we can conclude that the existence of relapse phenomena reduces the standard basic reproduction number $R_{0}^{\mathrm{standard}}$. This reduction was due to the dormant period experienced by infected individuals in the *hypnozoite* phase, which made them unable to directly infect healthy mosquitoes. As previously mentioned, malaria infection by *Plasmodium Vivax* and *Ovale* can result in a long dormant period of up to 2–3 years.
4. **Model with recrudescence only.** When relapse and reinfection are not involved in the original model (Equation (S1) in the Supplementary File), then we have $\bar{\beta_{2}}=0,\bar{\eta }=0$. Hence, the transmission diagram in Figure 1 is reduced to the one in Figure 7.
  The basic reproduction number of the non-dimensional model based on transmission diagram in Figure 7 is given by
  (9) $$\begin{array}{ccc} R_{0}^{\mathrm{recrudescence}} & = & \frac{\xi \;\left(\alpha \;\beta_{3}+\gamma \;\beta_{4}+\beta_{3}\mu_{h}\right)\beta_{1}}{\left(\xi +\mu_{h}\right)\left(\mu_{h}+1\right)\left(\alpha \;\gamma \;\left(1-\kappa_{2}\right)+\alpha \;\mu_{h}+\gamma \;\mu_{h}+{\mu_{h}}^{2}\right)\mu_{v}}\\ & = & \underset{\mathrm{effect of recrudescence}}{\underset{︸}{\left\{\frac{\alpha \;\gamma +\alpha \;\mu_{h}+\gamma \;\mu_{h}+{\mu_{h}}^{2}}{\alpha \;\gamma \;\left(1-\kappa_{2}\right)+\alpha \;\mu_{h}+\gamma \;\mu_{h}+{\mu_{h}}^{2}}\right\}}}\;\;\;R_{0}^{\mathrm{standard}}. \end{array}$$
  It can be seen that since $\frac{\alpha \;\gamma +\alpha \;\mu_{h}+\gamma \;\mu_{h}+{\mu_{h}}^{2}}{\alpha \;\gamma \;\left(1-\kappa_{2}\right)+\alpha \;\mu_{h}+\gamma \;\mu_{h}+{\mu_{h}}^{2}}>1$, we may conclude that recrudescence will increase the standard basic reproduction number.

To summarize the biological meaning of the above special cases of the basic reproduction number, we have the following relations:

1. The basic reproduction number when all recurrence phenomena are constructed as a multiplication between infection in humans, infection in mosquitoes, lifetime period of class *e*, and the ratio between the in- and outflow of class *l*. We call this basic reproduction number the standard basic reproduction number.
2. The existence of reinfection phenomena does not change the size of the standard basic reproduction number. It means that increasing reinfection the rate will not affect the size of the standard basic reproduction number. However, it will increase the endemic size and the possibility for the existence of multiple endemic conditions in the environment. We discuss this in the next section of this article.
3. The existence of relapse phenomena will reduce the size of the standard basic reproduction number. This is highly related to the duration of the dormant period of the *hypnozoite* inside the human body.
4. The existence of recrudescence phenomena will increase the size of the standard basic reproduction number.

### 3.3. Existence of the Endemic Equilibrium

In this section, we analyze the existence of the non-trivial equilibrium points, namely the malaria-endemic equilibrium point. This equilibrium differs from the malaria-free equilibrium, wherein malaria-endemic equilibria coexist between all classes, which means that malaria may still exist among the human and mosquito populations. The malaria-endemic equilibrium is given by

(10) $$\begin{array}{c} E_{2}=\left(s^{*},\;e^{*},\;d^{*},\;l^{*},\;i^{*},\;m^{*},\;v^{*}\right), \end{array}$$

where

$$\begin{array}{ccc} s^{*} & = & \frac{\left(\mu_{h}+1\right)\left(\xi +\mu_{h}\right)\left(\beta_{2}v^{*}+\eta +\mu_{h}\right)l^{*}}{\xi \;\beta_{1}\left(\beta_{2}v+\kappa_{1}\mu_{h}+\eta \right)v^{*}},\\ e^{*} & = & \frac{\left(\mu_{h}+1\right)\left(v^{*}\beta_{2}+\eta +\mu_{h}\right)l^{*}}{\xi \;\left(\beta_{2}v^{*}+\kappa_{1}\mu_{h}+\eta \right)},\\ d^{*} & = & \frac{\left(1-\kappa_{1}\right)\left(\mu_{h}+1\right)l^{*}}{v^{*}\beta_{2}+\kappa_{1}\mu_{h}+\eta },\\ l^{*} & = & \frac{\left(i^{*}\;\alpha \;\omega \mu_{h}+i^{*}\;\omega {\mu_{h}}^{2}+\alpha \;\gamma \;\left(1-\kappa_{2}\right)+\mu_{h}\alpha +\gamma \;\mu_{h}+{\mu_{h}}^{2}\right)i^{*}}{\left(\alpha +\mu_{h}\right)\left(1+\omega \;i^{*}\right)},\\ m^{*} & = & \frac{\gamma \;i^{*}}{\left(\alpha +\mu_{h}\right)\left(1+\omega i^{*}\right)},\\ v^{*} & = & \frac{\left(\alpha \;\omega \beta_{3}+\omega \beta_{3}\mu_{h}\right){i^{*}}^{2}+\left(\alpha \;\beta_{3}+\gamma \;\beta_{4}+\beta_{3}\mu_{h}\right)i^{*}}{\left(\alpha \;\omega \beta_{3}+\omega \beta_{3}\mu_{h}\right){i^{*}}^{2}+\left(\alpha \;\omega \mu_{v}+\omega \mu_{h}\mu_{v}+\alpha \;\beta_{3}+\gamma \;\beta_{4}+\beta_{3}\mu_{h}\right)i^{*}+\alpha \;\mu_{v}+\mu_{h}\mu_{v}}, \end{array}$$

and $i^{*}$ is a positive solution of the following five-degree polynomial:

(11) $$\begin{array}{ccc} f(i) & = & b_{6}\;i^{5}+b_{5}\;i^{4}+b_{4}\;i^{3}+b_{3}\;i^{2}+b_{2}\;i+b_{1}=0 \end{array}$$

with

$$\begin{array}{ccc} b_{6} & = & \omega^{3}{\beta_{3}}^{2}\left(\mu_{h}+1\right){\left(\alpha +\mu_{h}\right)}^{3}\left(\xi +\mu_{h}\right)\left(\mu_{h}+\phi \right)\left(\beta_{2}+\eta +\mu_{h}\right)\left(\beta_{1}+\mu_{h}\right),\\ b_{1} & = & \left(\xi +\mu_{h}\right)\left(\eta +\mu_{h}\right)\left(\mu_{h}+1\right)\left(\alpha \;\gamma \;\left(1-\kappa_{2}\right)+\mu_{h}\alpha +\gamma \;\mu_{h}+{\mu_{h}}^{2}\right)\mu_{v}\;\left(1-R_{0}\right). \end{array}$$

while $b_{2},b_{3},b_{4},$ and $b_{5}$ can not be shown in this article due to the complexity of their forms. Since $b_{6}>0$ and $b_{1}<0\Leftrightarrow R_{0}>1$, we always have at least one malaria-endemic equilibrium point if $R_{0}>1$. Furthermore, since the malaria-endemic equilibrium point is taken from a five-degree polynomial, the possibility that the malaria-endemic equilibrium point is not unique must be considered. For this purpose, we find that our model may have an endemic equilibrium even though $R_{0}<1$. Furthermore, we also have shown that it is possible to find one or three malaria-endemic points when $R_{0}>1$, and none or two malaria-endemic equilibrium points when $R_{0}<1$. Please see Supplementary Section S2 for the mathematical details of these results.

## 4. Bifurcation Analysis

The backward bifurcation phenomenon is believed to be one of the reasons why several types of diseases with relapse or reinfection periods are difficult to control. Many authors have studied a backward bifurcation phenomenon in the malaria model. In the non-dimensional version of our proposed malaria model, our previous analysis shows the possibility of the existence of backward bifurcation, which is indicated by the existence of an endemic equilibrium when $R_{0}<1$ under a specific condition (please see Supplementary Section S2). Epidemiologically, this means that the basic reproduction number can not be the only threshold in the determination of whether malaria exists or is extinct from the population.

In this section, our aim is to investigate the possible bifurcation phenomena in our model and determine another threshold that can be used to determine what type of bifurcation can arise depending on this new threshold. In order to do so, we use the well-known Castillo–Song bifurcation theorem [30]. For further details on the analysis of the bifurcation type from the non-dimensionalized malaria model, please see Supplementary Section S3. From the analysis that has been performed, we find that our model may undergo a backward bifurcation phenomenon at $R_{0}=1$.

The appearance of backward bifurcation phenomena (see Supplementary Section S3) indicates another threshold that can determine the existence or extinction of malaria from the field other than $R_{0}$, namely the $\omega^{*}$. The emergence of the backward bifurcation phenomenon indicates that concluding the basic reproduction number as the main threshold in malaria control is no longer sufficient. This is because even though the basic reproduction number is already less than one, it is still possible that malaria is endemic in the community. In the following paragraph, we discuss this theorem’s mathematical and biological consequences, backward and forward bifurcation, and hysteresis phenomena.

**Figure 8:**
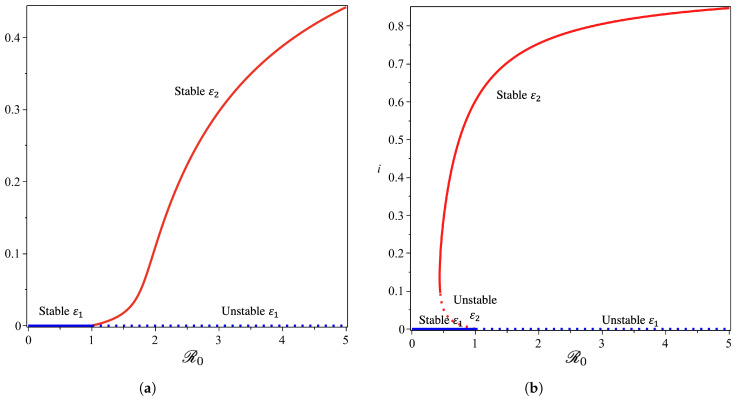
Forward (**a**) and backward (**b**) bifurcation phenomena of the non-dimensionalized malaria model (see Supplementary Section S1, Equation (S4)) from near $R_{0}=1$ with ω=25 and ω=85, respectively.

**Figure 9:**
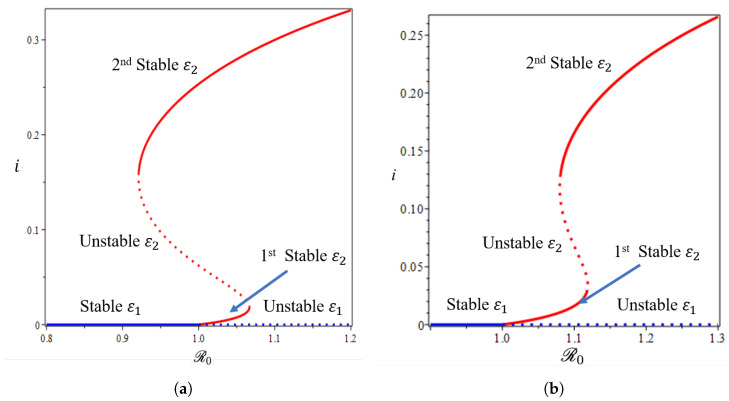
The 1st and 2nd types of forward bifurcation with hysteresis phenomena of the non-dimensionalized malaria model (**a**,**b**) (see Supplementary Section S1, Equation (S4)) from near $R_{0}=1$ with ω=38 and ω=34, respectively.

From the Castillo–Song bifurcation coefficient in Supplementary Section S3, we can see that the sign of A will determine the type of bifurcation that will appear from our model at $R_{0}=1$. When A<0, we will have a forward bifurcation phenomenon. On the other hand, when A>0, backward bifurcation is the phenomenon that we will obtain. To visualize this result, we use the parameter in Table 1, we calculate $\omega^{*}$, and we obtain $\omega^{*}=65.64$. We choose four different values of ω, and the bifurcation diagrams are shown in Figure 8 and Figure 9.

From Figure 8, we show that the malaria-free equilibrium point is always stable when $R_{0}<1$ and unstable when $R_{0}>1$. When the saturation parameter ω, which presents the capacity of the hospital, is more significant than $\omega^{*}$, then backward bifurcation arises (Figure 8b). This indicates that malaria does not go extinct, even when $R_{0}$ is already smaller than one. In this case, we have a large size of the malaria-endemic equilibrium that is also stable for some values of $R_{0}<1$. When the number of the bed capacity is significant in the hospital (presented by a much smaller value of ω compared to $\omega^{*}$), then forward bifurcation arises at $R_{0}=1$. Interesting results are shown in Figure 9. Even though we set the value of ω smaller than $\omega^{*}$, which will give us a forward bifurcation phenomenon, as we can see from Figure 9a that we may still have a stable endemic equilibrium for some values of $R_{0}<1$. The reason for this is that the forward bifurcation phenomena arise coupled with a hysteresis. As a result, we can have multiple stable malaria-endemic equilibria for some values of $R_{0}>1$, and bistability between the malaria-free equilibrium and the malaria-endemic equilibrium for some values of $R_{0}<1$.

In comparison to the simulation in Figure 9a where the bistability between the malaria-free and malaria-endemic equilibrium points could arise when $R_{0}<1$, the result in Figure 9b is different. We can see from Figure 9b that we never had bistability between malaria-free and malaria-endemic equilibria. We only obtain bistability between two malaria-endemic equilibrium points for some value of $R_{0}>1$. The phenomena above give an indication of the difficulty of creating malaria-controlling policy if only relying on the magnitude of the basic reproduction numbers. The emergence of a stable endemic point is still possible when backward bifurcation occurs because the basic reproduction number can lead to policies that are not optimal. Furthermore, the emergence of forward bifurcation with hysteresis allows the emergence of a stable endemic point of a large size even though the basic reproduction number is larger but close to one.

**Figure 10:**
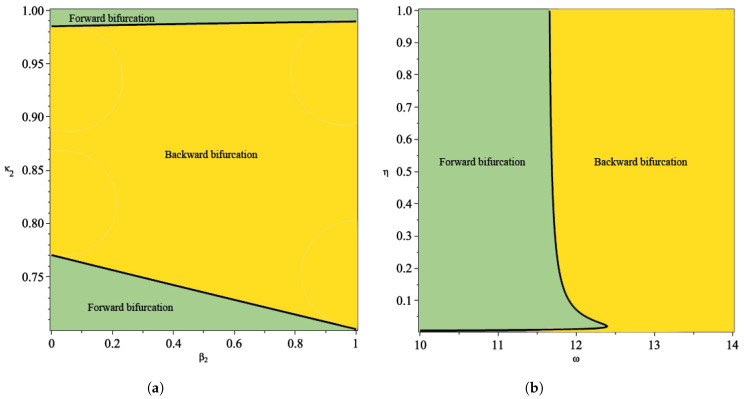
Plot of A as a function between rate of reinfection in human $\left(\beta_{2}\right)$ and recrudescence proportion $\left(\kappa_{2}\right)$ in (**a**) and between treatment saturation rate and rate of relapse (η) (**b**).

From the illustration of the impact of hospital capacity on the type of bifurcation of our model in Figure 8 and Figure 9, we can see that a smaller bed capacity in the hospital (larger ω) will trigger the appearance of backward bifurcation. Hence, more bed capacity in the hospital is better for malaria eradication policy. Furthermore, our aim in this article is also to find the impact of reinfection $\left(\beta_{2}\right)$, relapse (η), and recrudescence $\left(\kappa_{2}\right)$ on the type of bifurcation on our proposed model. To achieve this aim, we simulate the impact of $\beta_{2},\eta ,\omega$, and $\kappa_{2}$ on the sign of A. If A>0, then backward bifurcation appears, and if A<0, then forward bifurcation appears. Using the same parameter as shown in Table 1, the results are given in Figure 10. From Figure 10a, the larger the proportion of treated individuals that failed the treatment (larger $\kappa_{2}$), then the higher the chance that our model exhibits a backward bifurcation. On the other hand, there is an interval for $\beta_{2}$ depending on the value of $\kappa_{2}$ such that we can have a forward or backward bifurcation in our model. This means that a larger reinfection rate does not always trigger a backward bifurcation phenomenon. Figure 10b shows the impact of the treatment saturation parameter (ω) and relapse rate (η) on the bifurcation type of the proposed model. A smaller capacity of beds in the hospital (larger ω) will increase the chance of the appearance of backward bifurcation. On the other hand, with a larger relapse rate value, there exists a larger possibility that we can avoid a backward bifurcation. This illustration shows that relapse holds an important role in determining whether malaria will exist or become extinct when the basic reproduction number is already less than one, since it will trigger a condition where a basic reproduction number smaller than one will not guarantee the extinction of malaria.

## 5. Global Sensitivity Analysis

We perform a global sensitivity analysis using the combination of Latin hypercube sampling (LHS) and partial rank correlation coefficient (PRCC) [53,54] to determine the influential parameters of the model as in [55,56,57]. We measure against the increasing number of infected individuals which is

(12) $$I\left(t\right)=\int_{0}^{t_{f}}\left(\kappa_{2}\tilde{\alpha }T+\tilde{\epsilon }L\right)dt.$$

**Figure 11:**
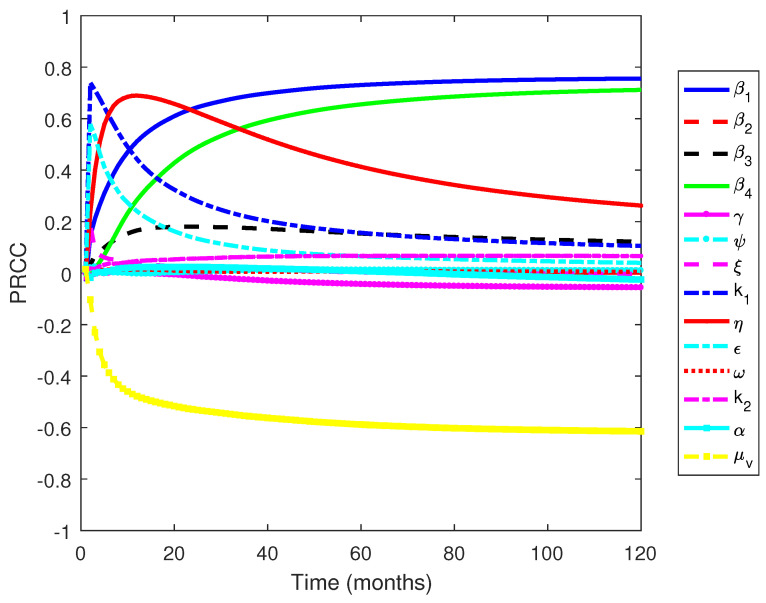
PRCC values when measured against an increasing number of infected individuals over time.

Figure 11 shows that the parameters $\beta_{1}$, $\beta_{4}$, and $\mu_{v}$ are the most influential parameters, where the first two have positive relationships and the latter has a negative relationship. The results are similar to the other work, where the transmission-related parameters and the death rate of mosquitoes are the most influential parameters [55]. However, our results provide detailed information regarding which transmission parameters govern the dynamics of infected individuals: the transmission rate from mosquito to susceptible humans ($\beta_{1}$) and the transmission rate from human to mosquitoes ($\beta_{4}$). On the other hand, the influence of the transmission rate from mosquitoes to dormant individuals is not as strong as the previously mentioned transmission parameters. The results indicate that the transmission from mosquitoes to susceptible human plays an essential role in determining the dynamics of malaria transmission. Furthermore, the death rate of mosquitoes, which are generally influenced by climatic factors, can reduce the number of infected individuals. This suggests that further work to analyze the effects of climatic factors is important and is the subject of future work.

## 6. Optimal Control Problem

### 6.1. Characterization of the Optimal Control Problem

In this section, we modify our proposed model in Equation (S1) of the Supplementary File to include three different interventions which depend on time, namely the use of bed net $\left(u_{1}\left(t\right)\right)$, hospitalization $\left(u_{2}\left(t\right)\right)$, and fumigation $\left(u_{3}\left(t\right)\right)$.

1. **Use of a bed net.** Use of bed net is reportedly successful in reducing malaria incidence worldwide [58]. Bed nets provide protection to humans from the bite of a mosquito. Let us assume $u_{1}$ represents the proportion of the human population who use a bed net. Hence, $u_{1}N$ and $(1-u_{1})N$ represent the total human population who use and do not use a bed net, respectively. The successful transmission rate for humans who use bed nets now read as $\delta {\bar{\beta }}_{i}$, where 1−δ presents the efficacy of bed nets in reducing the number of successful bites. Note that in δ∈(0,1), a smaller δ represents a bed net with better quality. Based on this assumption, the total number of new infections for non-users of bed nets in susceptible populations is given by
  $$f_{1}\left(u_{1},{\bar{\beta }}_{1}\right)={\bar{\beta }}_{1}\times \frac{(1-u_{1})S}{N}\times V=\left(1-u_{1}\right){\bar{\beta }}_{1}\frac{SV}{N},$$
  and the total number of new infections for the bed net users is given by
  $$f_{2}\left(u_{1},{\bar{\beta }}_{1}\right)=\delta {\bar{\beta }}_{1}\times \frac{u_{1}S}{N}\times V=\delta u_{1}{\bar{\beta }}_{1}\frac{SV}{N}.$$
  Therefore, total of new infections in the susceptible population is given by
  (13) $$f\left(u_{1},{\bar{\beta }}_{1}\right)=f_{1}\left(u_{1},\beta_{1}\right)+f_{2}\left(u_{1},\beta_{1}\right)=\left[\left(1-u_{1}\right)+u_{1}\delta \right]{\bar{\beta }}_{1}\frac{SV}{N}.$$
  Note that if the entire human population used a bed net and the quality of the bed net could provide 100% protection against mosquitoes’ bites (δ=0), then no infections would occur in the field (in this case, we have $f(u_{1},\beta_{1})=0$). On the other hand, if all humans used a bed net but the efficacy level (protection from mosquito bites) is not 100%, then there is still a possibility that new infections occur, which given by
  $$f\left(u_{1},\beta_{1}\right)=\delta {\bar{\beta }}_{1}\frac{SV}{N}.$$
  Furthermore, when not all humans use a bed net, but the efficacy level of the bed net is 100%, then the total number of new infections is given by
  $$f\left(u_{1},{\bar{\beta }}_{1}\right)=\left(1-u_{1}\right){\bar{\beta }}_{1}\frac{SV}{N}.$$
  A similar approach is applied to the reinfection term and the new infection in mosquito population term, which involve ${\bar{\beta }}_{2},{\bar{\beta }}_{3},$ and ${\bar{\beta }}_{4}$. Note that when $u_{1}=1$, the infection term is reduced into the standard model in Equation (S1) of the Supplementary File.
  For the sake of simplification, we use another interpretation of bed net use, the term $\left(\left(1-u_{1}\right)+u_{1}\delta \right)$, as follows. The term $\left(\left(1-u_{1}\right)+u_{1}\delta \right)$ can be rewritten as $\left[1-u_{1}\left(1-\delta \right)\right]=\left(1-u_{1}\zeta \right)$ where ζ=1−δ represents the effective bed net utilization rate. If ζ=0 or (equivalently) δ=1, then the bed net is useless, and regardless of the number of people who use the bed net, there will not be any impact on malaria prevention. In contrast, if ζ=1, which is equivalent to the condition δ=0, then the bed net can always provide protection to humans from mosquito bites. The larger the utilization rate $u_{1}\zeta$, the stronger the impact of bed net usage in the malaria prevention program. Therefore, instead of using the expression as in (13), we use the following expression to show the impact of bed net usage
  (14) $$f\left(u_{1},\beta_{1}\right)=\left(1-u_{1}\zeta \right){\bar{\beta }}_{1}\frac{SV}{N}.$$
  For another modeling approach on the application of bed nets in malaria intervention, please see [17,59].
2. **Hospitalization.** In endemic malaria areas, hospitalization is the most frequently used outbreak control effort. However, this effort is difficult to execute continuously at a high intensity. Therefore, instead of using the constant hospitalization rate of γ, we use the new term $u_{2}\left(t\right)$, which represents the time-dependent treatment rate.
3. **Fumigation.** For many types of vector-borne disease, including malaria, vector control programs are the most common intervention to control the spread of the disease. Hence, we introduce $u_{3}\left(t\right)$ as the additional death rate of the mosquito population due to fumigation, where the intervention depends on time.

Based on the above descriptions of three time-dependent interventions, the original model in Equation (S1) of the Supplementary File now reads as shown in Equation (S19) of the Supplementary Section S4. Our aim is to minimize the number of infected humans with an optimal intervention (as low as possible). Mathematically, the task reads as minimizing the following objective functional:

(15) $$J\left(u_{1},u_{2},u_{3}\right)=\int_{0}^{t_{f}}\left(\omega_{1}E+\omega_{2}D+\omega_{3}L+\omega_{4}I+\varphi_{1}u_{1}^{2}+\varphi_{2}u_{2}^{2}+\varphi_{2}u_{3}^{2}\right)dt,$$

where $\omega_{1},\;\omega_{2},\;\omega_{3}$, and $\omega_{3}$ are the weights of the objective function for E,D,L, and *I*, respectively. Furthermore, $\varphi_{j}$ for i=1,2,3 are the weight parameters for the control variables. $\omega_{i}$ and $\varphi_{j}$ are the weight parameters that can balance each term in J. We use the quadratic cost function for J to describe the cost of control efforts. quadratic form is a common way to express cost functions in many mathematical epidemiology models with optimal control [4,17]. This quadratic function can describe a nonlinear cost increase related to the implementation of control efforts in the field.

### 6.2. Optimal Control Characterization

Using Pontryagin’s maximum principle [60], the optimal solutions of $u_{1}$, $u_{2}$, and $u_{3}$ are given by

(16) $$\begin{array}{ccc} u_{1}^{*} & = & \min\left\{1,\max\left(0,\frac{\beta_{1}SV\zeta }{2\varphi_{1}N}\left(\lambda_{2}-\lambda_{1}\right)+\frac{\beta_{2}DV\zeta }{2\varphi_{1}N}\left(\lambda_{4}-\lambda_{3}\right)+\left(\frac{\beta_{3}UI\zeta +\beta_{2}UT\zeta }{2\varphi_{1}N}\right)\left(\lambda_{9}-\lambda_{8}\right)\right)\right\},\\ u_{2}^{*} & = & \min\left\{1,\max\left(0,\frac{I}{2\varphi_{2}\left(1+\omega I\right)}\left(\lambda_{5}-\lambda_{6}\right)\right)\right\},\\ u_{3}^{*} & = & \min\left\{1,\max\left(0,\frac{U\lambda_{8}+V\lambda_{9}}{2\varphi_{3}}\right)\right\}. \end{array}$$

Please see the Supplementary Section S4 for details on the derivation of the optimal control characterization.

### 6.3. Optimal Control Simulation

This section presents the numerical optimal control simulation to numerically solve the model with control and without control. Employing the backward and forward sweep as described in [61], we obtained the optimality of the model, which is comprised of the model without control, the adjoint system (see Supplementary Section S4), and the optimality conditions (17). To ascertain which strategy or combination gives the efficient methods of controlling malaria disease spread, we considered the following strategies/scenarios for our simulation results as enumerated below:

#### 6.3.1. **Strategy 1.** Single Intervention: Use of Bed Net Only

**Figure 12:**
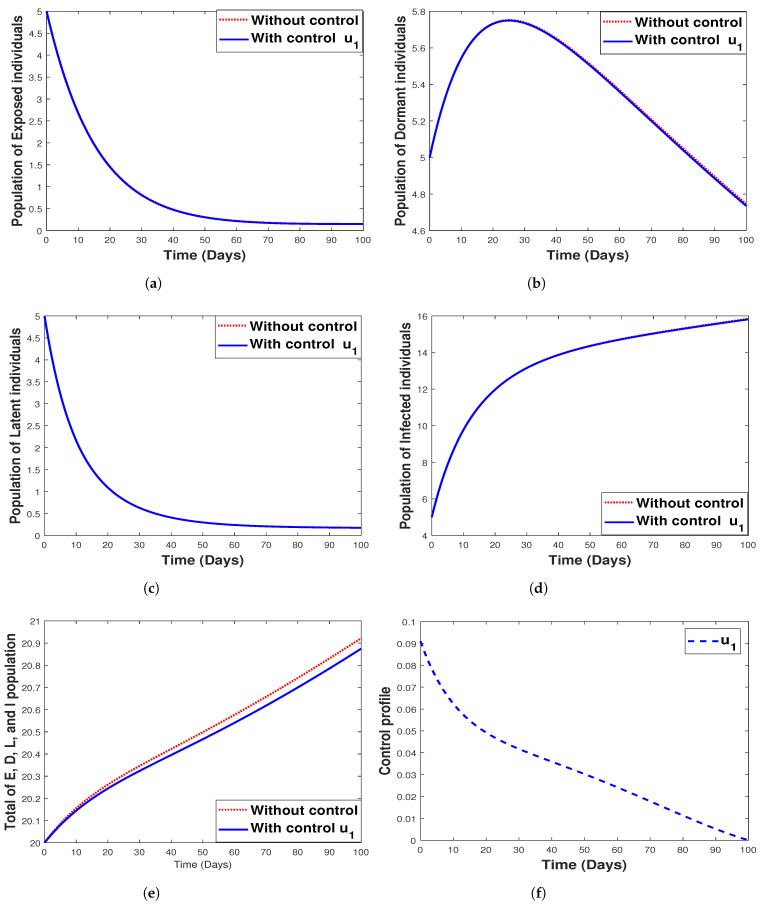
Simulation results for strategy 1 shows the dynamics of exposed compartment, dormant compartment, latent compartment, infected compartment, total of exposed, dormant, latent, and infected population, and the dynamic of control $u_{1}$ for subfigure (**a**)–(**f**), respectively.

The intervention of bed net usage only was simulated against all the model state variables, and the results are given in Figure 12. We observe in Figure 12 that the use of bed net as a control strategy only impacts the dormant population and the total population consisting of the exposed, dormant, latent, and infected individuals, while Figure 12a–d shows that the control $u_{1}$ does not have an impact on the control of the exposed, dormant, latent, and infected population, respectively. Figure 12 shows the control profile for strategy 1.

#### 6.3.2. **Strategy 2.** Single Intervention: Use of Hospitalization Only

**Figure 13:**
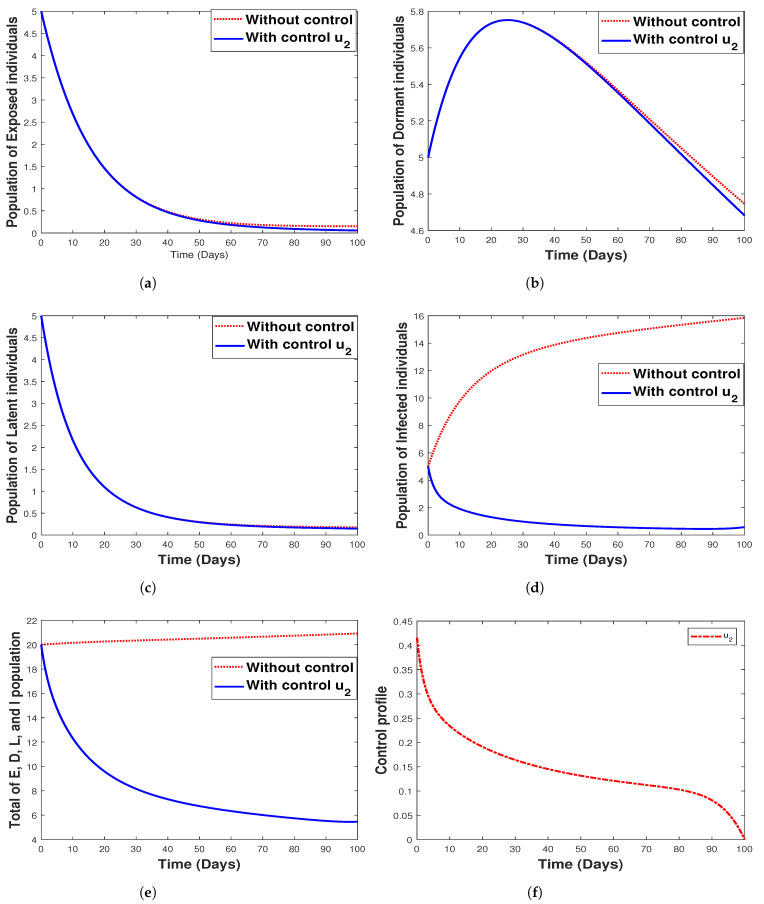
Simulation results for strategy 2 shows the dynamics of exposed compartment, dormant compartment, latent compartment, infected compartment, total of exposed, dormant, latent, and infected population, and the dynamic of control $u_{2}$ for subfigure (**a**)–(**f**), respectively.

The use of hospitalization as the only control measure when simulating the model with and without optimal control is depicted in Figure 13. We see that this control strategy has no impact on the exposed (Figure 13a) and latent (Figure 13c) population as well as the control profile, as indicated in Figure 13f. It can be seen that hospitalization has a negligible impact on the dormant individuals and significantly affects both infected humans and the total population (that is, the total population of E,D,L, and *I*) as depicted in Figure 13d,e. The biological implication of hospitalization, as seen in Figure 13c, shows a decrease in the number of infected individuals with an increase in time. In other words, there is a reduction in the number of humans infected with malaria disease.

#### 6.3.3. **Strategy 3.** Single Intervention: Use of Fumigation Only

**Figure 14:**
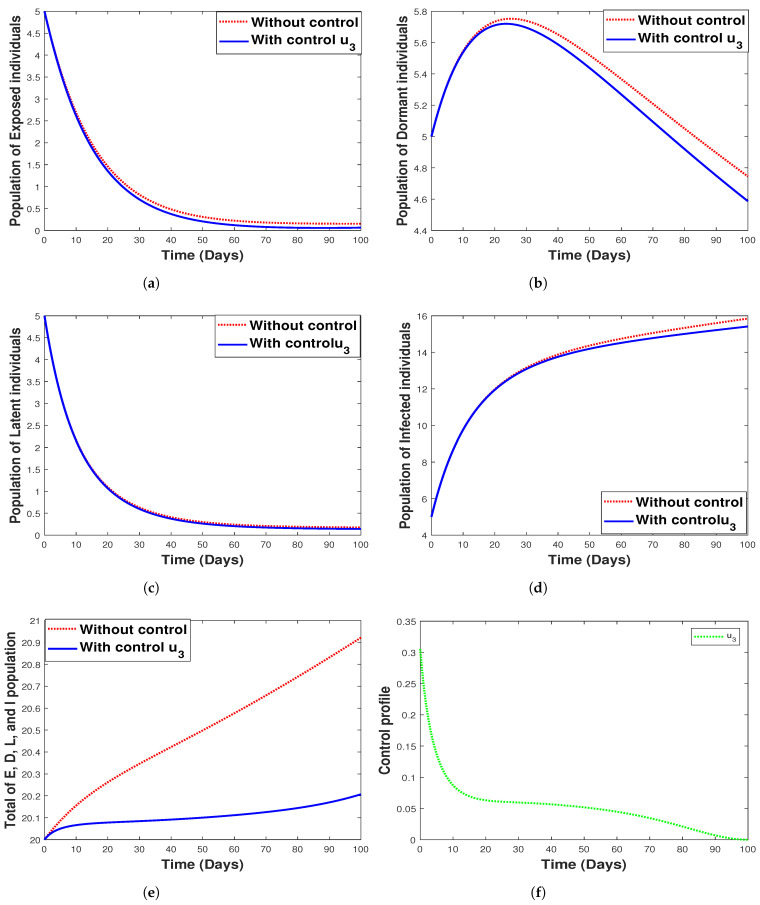
Simulation results for strategy 3 shows the dynamics of exposed compartment, dormant compartment, latent compartment, infected compartment, total of exposed, dormant, latent, and infected population, and the dynamic of control $u_{3}$ for subfigure (**a**)–(**f**), respectively.

Figure 14 shows the implementation of fumigation as the only control method with its control profile. The optimal control time series trajectories for the dormant population and the total combined population show a great decline in the number of individuals with an increase in time (See Figure 14b,e). Additionally, we observe that strategy 3 has a small impact on controlling exposed, infected, and latent individuals, as seen in Figure 14a,d,c, respectively.

#### 6.3.4. **Strategy 4.** Double Intervention: Combination of Hospitalization and Fumigation

**Figure 15:**
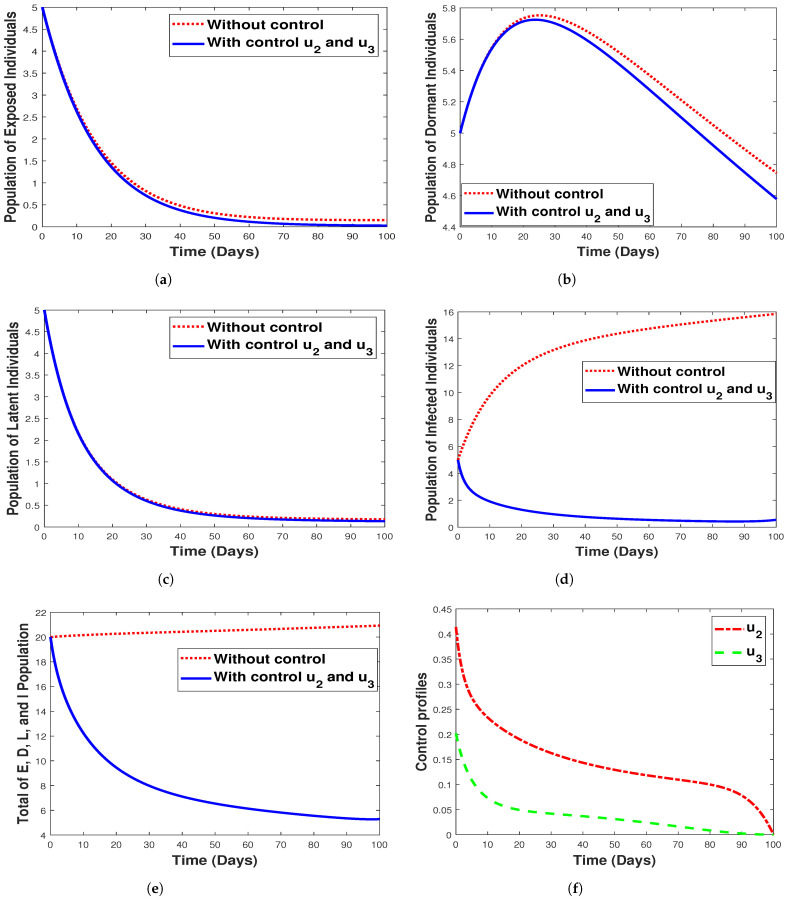
Simulation results for strategy 4 shows the dynamics of exposed compartment, dormant compartment, latent compartment, infected compartment, total of exposed, dormant, latent, and infected population, and the dynamic of control $u_{1}$ and $u_{2}$ for subfigure (**a**)–(**f**), respectively.

While Figure 15 shows the implementation of the combined strategy consisting of hospitalization and fumigation, Figure 15f shows its control profile. Clearly observable in Figure 15b,e, the implementation of a combined strategy reduced the number of the dormant, infectious, and the total combined populations while having a lesser impact on the exposed/latent individuals, as seen in Figure 15a,c. We can deduce that using the combined strategy is the best control measure to eliminate malaria infections. The shaded area in the control profile shows from our modeling that malaria can be controlled when $u_{2}=0.2$ and $u_{3}=0.4$, rather than when the values are equivalent. However, this signifies that the spread of malaria infection in any community with the disease can be prevented or eradicated by implementing both fumigation to control/decrease mosquito birth rate and hospitalization of infected individuals. Additionally, we observe that strategy 4 has a small impact in controlling exposed, infected, and latent individuals, as seen in Figure 15a,d,c respectively.

## 7. Cost-Effectiveness Analysis

Here, we present the cost-effectiveness analysis for the model with and without control, intending to determine the best control strategies among all the scenarios. The total averted infection and total cost for each scenario are given in Table 2. The costs of the scenarios are obtained from the numerical output for the objective function Equation (15), arranged in incremental order.

(17) $$\mathrm{ICER}=\frac{\mathrm{Difference}\;\mathrm{in}\;\mathrm{total}\;\mathrm{costs}\;\mathrm{by}\;\mathrm{strategies}\;A\;\mathrm{and}\;B}{\mathrm{Difference}\;\mathrm{in}\;\mathrm{the}\;\mathrm{total}\;\mathrm{number}\;\mathrm{of}\;\mathrm{averted}\;\mathrm{infection}\;\mathrm{by}\;\mathrm{strategies}\;A\;\mathrm{and}\;B}.$$

Using the values in Table 2, we calculate the incremental cost-effectiveness ratio (ICER) by comparing each successive strategy with each other by employing (17) as defined in [62].

We arrange the strategies in increasing order from the lowest to the highest ICER values (see Table 2). Note that in order to make decisions, higher ICER values are eliminated in each step [62]. First, we calculate and compare the ICER values of strategies 1 and 3, where the ICER for strategy 1 is calculated by comparing the baseline total averted infections and total cost as given below

$$\begin{array}{ccc} \mathrm{ICER}(1) & = & \frac{2.0481\times 10^{3}-0}{1.4963-0}=1368.7763\\ \mathrm{ICER}(3) & = & \frac{\left(2.0180-2.0481\right)\times 10^{3}}{(18.4577-1.4963}=-1.7746177. \end{array}$$

Considering the results from the calculations of ICER(1) and ICER(3), we reject ICER(1), as it has a higher cost than ICER(3). Next, we compute and compare the ICER values for strategies 3 and 2. Using the total averted infections and total cost for strategy 2, we find the new ICER value using strategy 2 as a baseline while comparing it to the value of ICER(3) as follows:

(18) $$\begin{array}{ccc} \mathrm{ICER}(3) & = & \frac{2.0180\times 10^{3}}{18.4577}=109.33101,\\ \mathrm{ICER}(2) & = & \frac{842.5959-2.0180\times 10^{3}}{\left(765.5569-18.4577\right)}=-1.573291. \end{array}$$

Based on the values in expression (18), ICER(2) is cheaper than ICER(3). Hence, we reject ICER(3). Continuing the iteration

(19) $$\begin{array}{ccc} \mathrm{ICER}(2) & = & \frac{842.5959}{765.599}=1.1006313182\\ \mathrm{ICER}(4) & = & \frac{\left(1.5202\right)\times 10^{6}-842.5959}{\left(773.8898-765.5569\right)}=182332.3698. \end{array}$$

Similarly, comparing ICER(2) and ICER(4) using Equation (19), we eliminate strategy 4 since strategy 2 is cheaper than strategy 4. We conclude that to reach elimination/control of malaria, it is most cost-effective to first implement strategy 2. We repeat the above iteration to help establish the next most cost-effective strategy. Using the simulation results from the re-computation, we find that strategy 3 is the subsequent most cost-saving intervention after strategy 2 followed by strategies 1 and 4. Our results suggest that strategy 4 is the least cost-effective intervention required for malaria control as projected by our modeling framework.

## 8. Discussion and Conclusions

There are many mathematical models discussing malaria transmission, such as [4,14,15,17,21,23] and many more, but none of the mentioned references discuss the impact of relapse, recrudescence, and reinfection all together in one model. Recently, the authors of [29] introduced a complex nine-dimensional system of ordinary differential equations model to describe malaria transmission under the impact of relapse, recrudescence, and reinfection. They found that backward bifurcation phenomena can be avoided in the absence of reinfection. Different than [29], here we introduced a mathematical model for malaria transmission considering the impact of relapse, reinfection, recrudescence, and limited hospital bed capacity which affect the recovery rate. Malaria incidence data from the Wee Luri district in Central Sumba, Kupang, Indonesia, were used to estimate the model parameters. We investigated the existence and the local stability of the malaria-free equilibrium point utilizing the concept of the basic reproduction number [50]. We found that malaria can be driven to extinction if the basic reproduction number is less than one and always exists if the basic reproduction number is larger than one. The malaria-endemic equilibrium can not be shown explicitly. A gradient analysis and Descartes’s rules of sign change were then used to show the malaria-endemic equilibrium when the basic reproduction number is larger than one. The concept of center manifold theory was used to establish the local stability criteria of the malaria-endemic equilibrium. Using the Castillo–Song bifurcation theorem [30], we determined another threshold other than basic reproduction number which can determine the existence or extinction of malaria from the system. Our analysis shows that our model may exhibit backward or forward bifurcation with hysteresis, triggering multiple malaria-endemic equilibria around the basic reproduction number equal to one. The existence of backward bifurcation makes malaria eradication more difficult since a basic reproduction number of less than one is no longer a sufficient indicator of the endemicity level.

From sensitivity analysis on the second threshold $\left(\omega^{*}\right)$ which may determine the type of bifurcation on our model, our findings suggest that backward bifurcation may likely occur given the following: (1) the hospital bed capacity is sufficiently small, (2) the relapse rate from dormant to latent compartment decreases, and (3) the proportion of treated individuals who failed the treatment is sufficiently large. Furthermore, we find that a more significant value of the reinfection rate cannot always guarantee the existence of a backward bifurcation phenomenon. We also studied the global sensitivity analysis of the total number of infected individuals (I). It was observed that the primary infection of humans and mosquitoes and the death rate of mosquitoes are the most influential parameters for determining the increase in the number of newly infected individuals. Hence, reducing the infection rate and increasing the mosquito’s death rate could be an option for reducing the number of infected individuals. Additionally, an increase in reinfection, recrudescence, and relapse increases the number of newly infected individuals.

Further, an optimal control problem was formulated by modifying the malaria model without control, incorporating three control variables: the use of a bed net, hospitalization, and fumigation. The model consists of these interventions as a time-dependent variable to minimize the number of infectious individuals and the cost of implementing the intervention strategy. The characterization of the optimal control problem was done using the Pontryagin maximum principle [60]. We solved our optimal control problem using the forward–backward iterative method until the convergence criteria are achieved. Numerical simulations for each control variable depict the fact that the use of bed net, hospitalization, and fumigation can decrease the spread of malaria (see Figure 12, Figure 13 and Figure 14. However, we can see from Figure 12 that the use of a bed net is insignificant in reducing the number of infectious individuals when compared to hospitalization and fumigation intervention. Hence, we combine hospitalization and fumigation as the fourth strategy, and the result can be seen in Figure 15. Our sensitivity analysis indicates that the combination of hospitalization and fumigation is the most cost-effective strategy. Implementing hospitalization is the most cost-effective strategy if a single intervention is preferable to a combination of two or more interventions. Another interesting observation is that if the number of infected individuals already decreases, then the intensity of the intervention could be reduced to obtain a lower implementation cost. Therefore, we may conclude that intervention with hospitalization, fumigation, and bed nets can be used as a critical intervention strategy for controlling malaria, although bed net use is not as effective as the other two interventions. Conclusively, we predict that implementing the interventions which comprise the simultaneous use of hospitalization, fumigation, and bed nets will play a crucial role in the control of malaria disease.

However, our model has some limitations. As mentioned by several authors [4,63], vector bias has an essential role in determining the success of malaria eradication programs. Another important factor that needs to be discussed is the seasonal impact on malaria transmission, as mentioned by many authors [64,65]. Including these factors in our model will be the future quest in order to obtain a better understanding of malaria transmission.

## Figures and Tables

**Table 1:** Description of parameters of the model (See Supplementary File, Equation (S1) for the model).

Par	Description	Units	Interval Values	Baseline Value	Ref.
$\Lambda_{h}$	Recruitment rate of human population	$\frac{\mathrm{human}}{\mathrm{day}}$	(0,∞)	$\frac{100,000}{60\times 365}$	Estimated
$\bar{\beta_{1}}$	Average probability of successful transmission rate from mosquito to human in *S*	$\frac{\mathrm{human}}{\mathrm{day}\times \mathrm{mosq}.}$	Fitted	0.062	Fitted
$\bar{\beta_{2}}$	Average probability of successful transmission rate from mosquito to human in *D* due to reinfection	$\frac{\mathrm{human}}{\mathrm{day}\times \mathrm{mosq}.}$	Fitted	0.06	Fitted
$\bar{\xi }$	Intrinsic incubation rate of *E*	$\frac{1}{\mathrm{day}}$	$\left[\frac{1}{30},\frac{1}{10}\right]$	$\frac{1}{15}$	[34,37]
$\bar{\epsilon }$	Transition from *L* to *D* after incubation period and ready to attack red blood cells	$\frac{1}{\mathrm{day}}$	$\left[\frac{1}{10},\frac{1}{3}\right]$	$\frac{1}{7}$	[34]
$\bar{\eta }$	Rate of relapse	$\frac{1}{\mathrm{day}}$	$\left[\frac{1}{5\times 365},\frac{1}{60}\right]$	$\frac{1}{9\times 30}$	[38,39]
$\kappa_{1}$	Proportion of exposed individuals who do not experience a dormant period	-	[0,1]	0.7	Estimated
$\bar{\mu_{h}}$	Natural human death rate	$\frac{1}{\mathrm{day}}$	$[\frac{1}{85\times 365},$ $\frac{1}{54\times 365}]$	$\frac{1}{60\times 365}$	[34]
$\bar{\gamma }$	Treatment rate	$\frac{1}{\mathrm{day}}$	(0,∞)	0.013	Fitted
$\bar{\alpha }$	Recovery rate	$\frac{1}{\mathrm{day}}$	$\left[\frac{1}{100},\frac{1}{10}\right]$	$\frac{1}{30}$	[27,40]
$\bar{\omega }$	Half-saturation parameter	$\frac{1}{\mathrm{human}}$	[0,1)	$\frac{1}{100}$	Estimated
$\kappa_{2}$	Proportion of treated individuals who experience recrudescence (treatment failure)	-	[0,0.2]	0.19	[41,42]
$\bar{\phi }$	Rate of loss of natural immunity in human population	$\frac{1}{\mathrm{day}}$	$\left[5.5,110\right]\times 10^{-4}$	$2.7\times 10^{-3}$	[43]
$\Lambda_{v}$	Recruitment rate of mosquito population	$\frac{\mathrm{mosq}.}{\mathrm{day}}$	$1.5\times 10^{5}\left[\frac{1}{21},\frac{1}{7}\right]$	$\frac{1.5}{21}\times 10^{5}$	Estimated
$\bar{\beta_{3}}$	Average probability of successful transmission rate in mosquito after biting *I* individuals	$\frac{1}{\mathrm{day}}$	Fitted	0.048	Fitted
$\bar{\beta_{4}}$	Average probability of successful transmission rate in mosquito after biting *T* individuals	$\frac{1}{\mathrm{day}}$	Fitted	0.048	Fitted
$\bar{\mu_{v}}$	Natural mosquitoes’ death rate	$\frac{1}{\mathrm{day}}$	$\left[\frac{1}{21},\frac{1}{7}\right]$	$\frac{1}{21}$	[44]

**Table 2:** Numerical value of total averted infections and cost of each strategy.

Strategies	Optimal Controls	Total Averted Infection	Total Cost
1	$u_{1}^{*}$	1.4963	$2.0481\times 10^{3}$
3	$u_{3}^{*}$	18.4577	$2.0180\times 10^{3}$
2	$u_{2}^{*}$	765.5569	842.5959
4	$u_{2}^{*},u_{3}^{*}$	773.8898	$1.5202\times 10^{6}$

## Data Availability

The data presented in this study are available on request from the corresponding author.

## Supplementary Materials

The following supporting information can be downloaded at: https://www.mdpi.com/article/10.3390/tropicalmed7100263/s1.
