# Supplementary material for: Assessing the Impact of Relapse, Reinfection and Recrudescence on Malaria Eradication Policy: A Bifurcation and Optimal Control Analysis
Source: Trop Med Infect Dis. 2022 Sep 24;7(10):263. doi: 10.3390/tropicalmed7100263 (PMC9609641; doi:10.3390/tropicalmed7100263)
Supplement: Supplementary file 1 [file tropicalmed-07-00263-s001.zip › tropicalmed-1820060-Supplementary.pdf]

Assessing the impact of relapse, reinfection and recrudescence on malaria eradication policy : A bifurcation and optimal control analysis

Supplementary Materials

S. 1 Non–dimensionalization process of malaria model The original model for malaria transmission in this article is given by:

$$\begin{aligned}
\frac{dS}{dt} &= \Lambda_h - \frac{\bar{\beta}_1 SV}{N} - \bar{\mu_h} S + \bar{\phi}\, R, \\
\frac{dE}{dt} &= \frac{\bar{\beta}_1 SV}{N} - \bar{\xi}\, E - \bar{\mu_h} E, \\
\frac{dD}{dt} &= (1-\kappa_1)\,\bar{\xi}\, E - \frac{\bar{\beta}_2 DV}{N} - \bar{\eta}\, D - \bar{\mu_h} D, \\
\frac{dL}{dt} &= \kappa_1 \bar{\xi}\, E + \frac{\bar{\beta}_2 DV}{N} + \bar{\eta}\, D - \bar{\epsilon}\, L - \bar{\mu_h} L, \\
\frac{dI}{dt} &= \kappa_2\, \bar{\alpha}\, T + \bar{\epsilon}\, L - \frac{\bar{\gamma}\, I}{1+\bar{\omega}\, I} - \bar{\mu_h} I, \\
\frac{dT}{dt} &= \frac{\bar{\gamma}\, I}{1+\bar{\omega}\, I} - \bar{\alpha}\, T - \bar{\mu_h} T, \\
\frac{dR}{dt} &= (1-\kappa_2)\ \bar{\alpha}\, T - \bar{\phi}\, R - \bar{\mu_h} R, \\
\frac{dU}{dt} &= \Lambda_v - \frac{\bar{\beta}_3 UI}{N} - \frac{\bar{\beta}_4\, U\, T}{N} - \bar{\mu_v} U, \\
\frac{dV}{dt} &= \frac{\bar{\beta}_3 UI}{N} + \frac{\bar{\beta}_4\, U\, T}{N} - \bar{\mu_v} V.
\end{aligned} \tag{1}$$

All parameters are assumed to be positive. Please note that system 1 equipped with a non-negative initial conditions. Summing the first seven equation in system (1), we have that the dynamic of total of human population is given by

$$\begin{aligned}
\frac{dN}{dt} &= \frac{dS}{dt} + \frac{dE}{dt} + \frac{dD}{dt} + \frac{dL}{dt} + \frac{dI}{dt} + \frac{dT}{dt} + \frac{dR}{dt} \\
&= \Lambda_h - \bar{\mu_h}\,(S+E+D+L+I+T+R) \\
&= \Lambda_h - \bar{\mu_h} N.
\end{aligned}$$

In addition, we have that the total of mosquito population is given by

$$\frac{dM}{dt} = \frac{dU}{dt} + \frac{dV}{dt}$$

$$
\begin{aligned}
&= \Lambda_v - \bar{\mu_v}\,(U+V) \\
&= \Lambda_v - \bar{\mu_v} M.
\end{aligned}
$$

Assuming total population is always constant at N, and the following transformations on parameter and variable,

$$
\begin{aligned}
& s=\frac{S}{N};\;\; e=\frac{E}{N};\;\; d=\frac{D}{N};\;\; l=\frac{L}{N};\;\; i=\frac{I}{N};\;\; m=\frac{T}{N};\;\; r=\frac{R}{N};\;\; u=\frac{U}{M};\;\; v=\frac{V}{M}; \\
& \tau=\bar{\epsilon}t;\;\; \beta_1=\frac{\bar{\beta_1}}{\bar{\epsilon}}\frac{M}{N};\;\; \beta_2=\frac{\bar{\beta_2}}{\bar{\epsilon}}\frac{M}{N};\;\; \beta_3=\frac{\bar{\beta_3}}{\bar{\epsilon}};\;\; \beta_4=\frac{\bar{\beta_4}}{\bar{\epsilon}};\;\; \mu_h=\frac{\bar{\mu_h}}{\bar{\epsilon}};\;\; \phi=\frac{\bar{\phi}}{\bar{\epsilon}}; \\
& \omega=\bar{\omega}N;\;\; \xi=\frac{\bar{\xi}}{\bar{\epsilon}};\gamma=\frac{\bar{\gamma}}{\bar{\epsilon}};\;\; \alpha=\frac{\bar{\alpha}}{\bar{\epsilon}};\;\; \eta=\frac{\bar{\eta}}{\bar{\epsilon}};\;\; \mu_v=\frac{\bar{\mu_v}}{\bar{\epsilon}};
\end{aligned} \tag{2}
$$

we obtain the system for malaria model of system (1) in the term of frequencies where $s+e+d+l+i+m+r=1$, and $u+v=1$ as follows:

$$
\begin{aligned}
\frac{ds}{d\tau} &= \mu_h - \beta_1\, s\, v - \mu_h\, s + \phi\, r, \\
\frac{de}{d\tau} &= \beta_1\, s\, v - \xi\, e - \mu_h\, e, \\
\frac{dd}{d\tau} &= (1-\kappa_1)\;\xi\, e - \beta_2\, d\, v - \eta\, d - \mu_h\, d, \\
\frac{dl}{d\tau} &= \kappa_1\, \xi\, e + \beta_2\, d\, v + \eta\, d - l - \mu_h\, l, \\
\frac{di}{d\tau} &= \kappa_2\, \alpha\, m + l - \frac{\gamma\, i}{1+\omega\, i} - \mu_h\, i, \\
\frac{dm}{d\tau} &= \frac{\gamma\, i}{1+\omega\, i} - \alpha\, m - \mu_h\, m, \\
\frac{dr}{d\tau} &= (1-\kappa_2)\;\alpha\, m - \phi\, r - \mu_h\, r, \\
\frac{du}{d\tau} &= \mu_v - \beta_3\, i\, u - \beta_4\, m\, u - \mu_v\, u, \\
\frac{dv}{d\tau} &= \beta_3\, i\, u + \beta_4\, m\, u - \mu_v\, v.
\end{aligned} \tag{3}
$$

Since $r = 1-s-e-d-l-i-m$, and $u = 1-v$, then we have the following 7-dimensional system which dynamically equivalent with system (3) :

$$
\begin{aligned}
\frac{ds}{d\tau} &= \mu_h - \beta_1\, s\, v - \mu_h\, s + \phi\,(1-s-e-d-l-i-m), \\
\frac{de}{d\tau} &= \beta_1\, s\, v - \xi\, e - \mu_h\, e, \\
\frac{dd}{d\tau} &= (1-\kappa_1)\;\xi\, e - \beta_2\, d\, v - \eta\, d - \mu_h\, d, \\
\frac{dl}{d\tau} &= \kappa_1\, \xi\, e + \beta_2\, d\, v + \eta\, d - l - \mu_h\, l, \\
\frac{di}{d\tau} &= \kappa_2\, \alpha\, m + l - \frac{\gamma\, i}{1+\omega\, i} - \mu_h\, i, \\
\frac{dm}{d\tau} &= \frac{\gamma\, i}{1+\omega\, i} - \alpha\, m - \mu_h\, m, \\
\frac{dv}{d\tau} &= \beta_3\, i\,(1-v) + \beta_4\, m\,(1-u) - \mu_v\, v.
\end{aligned} \tag{4}
$$

Theorem 1. *With non-negative initial condition $s>0, e\geq 0, d\geq 0, l\geq 0, i\geq 0, m\geq 0$ and $v\geq 0$, then any solution of system (4) will always be non-negative for all time $t\geq 0$.*

Proof. From the model in system (4), we obtain:

$$
\frac{ds}{d\tau}(s=0, e\geq 0, d\geq 0, l\geq 0, i\geq 0, m\geq 0, v\geq 0) \;=\; \mu_h + \pi(1-e-d-l-i-m) > 0,
$$

$$
\begin{aligned}
\frac{de}{d\tau}(s>0, e=0, d\geq 0, l\geq 0, i\geq 0, m\geq 0, v\geq 0) &= \beta_1 sv \geq 0,\\
\frac{dd}{d\tau}(s>0, e\geq 0, d=0, l\geq 0, i\geq 0, m\geq 0, v\geq 0) &= (1-\kappa_1)\xi e \geq 0,\\
\frac{dl}{d\tau}(s>0, e\geq 0, d\geq 0, l=0, i\geq 0, m\geq 0, v\geq 0) &= \kappa_1\xi e + \beta_2 dv + \eta d \geq 0,\\
\frac{di}{d\tau}(s>0, e\geq 0, d\geq 0, l\geq 0, i=0, m\geq 0, v\geq 0) &= \kappa_2\alpha m + l,\\
\frac{dm}{d\tau}(s>0, e\geq 0, d\geq 0, l\geq 0, i\geq 0, m=0, v\geq 0) &= \frac{\gamma i}{1+\omega i} \geq 0,\\
\frac{dv}{d\tau}(s>0, e\geq 0, d\geq 0, l\geq 0, i\geq 0, m\geq 0, v=0) &= \beta_3 i + \beta_4 m(1-u) \geq 0.
\end{aligned}
$$

The above rates for each variables are all non-negative on the boundary planes of the non-negative $\mathbb{R}_7^+$. Hence, we conclude that the direction of all vector fields are point inward in to the region. Therefore, if the initial condition is non-negative inside of $\mathbb{R}_7^+$, then the solution will always be non-negative for all $\tau > 0$. □

S. 2 Existence of the endemic equilibrium

The malaria-endemic equilibrium of of the malaria model (4) is given by

$$\mathcal{E}_2 = (s^*,\ e^*,\ d^*,\ l^*,\ i^*,\ m^*,\ v^*)\,, \tag{5}$$

where

$$\begin{aligned}
s^* &= \frac{(\mu_h+1)\,(\xi+\mu_h)\,(\beta_2 v^*+\eta+\mu_h)\,l^*}{\xi\,\beta_1\,(\beta_2 v+\kappa_1\mu_h+\eta)\,v^*},\\
e^* &= \frac{(\mu_h+1)\,(v^*\beta_2+\eta+\mu_h)\,l^*}{\xi\,(\beta_2 v^*+\kappa_1\mu_h+\eta)},\\
d^* &= \frac{(1-\kappa_1)\,(\mu_h+1)\,l^*}{v^*\beta_2+\kappa_1\mu_h+\eta},\\
l^* &= \frac{\left(i^*\,\alpha\,\omega\mu_h+i^*\,\omega{\mu_h}^2+\alpha\,\gamma\,(1-\kappa_2)+\mu_h\alpha+\gamma\,\mu_h+{\mu_h}^2\right)i^*}{(\alpha+\mu_h)\,(1+\omega\,i^*)},\\
m^* &= \frac{\gamma\,i^*}{(\alpha+\mu_h)\,(1+\omega i^*)},\\
v^* &= \frac{(\alpha\,\omega\beta_3+\omega\beta_3\mu_h)\,{i^*}^2+(\alpha\,\beta_3+\gamma\,\beta_4+\beta_3\mu_h)\,i^*}{(\alpha\,\omega\beta_3+\omega\beta_3\mu_h)\,{i^*}^2+(\alpha\,\omega\mu_v+\omega\mu_h\mu_v+\alpha\,\beta_3+\gamma\,\beta_4+\beta_3\mu_h)\,i^*+\alpha\,\mu_v+\mu_h\mu_v},
\end{aligned}$$

and i^* is a positive solution of the following 5-degree polynomial:

$$f(i) \;=\; b_6\,i^5+b_5\,i^4+b_4\,i^3+b_3\,i^2+b_2\,i+b_1=0 \tag{6}$$

with

$$\begin{aligned}
b_6 &= \omega^3{\beta_3}^2\,(\mu_h+1)\,(\alpha+\mu_h)^3\,(\xi+\mu_h)\,(\mu_h+\phi)\,(\beta_2+\eta+\mu_h)\,(\beta_1+\mu_h)\,,\\
b_1 &= (\xi+\mu_h)\,(\eta+\mu_h)\,(\mu_h+1)\left(\alpha\,\gamma\,(1-\kappa_2)+\mu_h\alpha+\gamma\,\mu_h+{\mu_h}^2\right)\mu_v\,(1-\mathscr{R}_0).
\end{aligned}$$

while b_2, b_3, b_4, and b_5 can not be shown in this article due to it complexity form. Based on the form of b_6 and b_1 on polynomial (6), we have the following theorem.

Theorem 2. *The malaria model* (4) *always has at least one malaria-endemic equilibrium when* $\mathscr{R}_0 > 1$.

Proof. Since b_6 as the coefficient of the highest order on $f(i)$, then we have $\lim_{i\to\infty} f(i) = \infty$ and $\lim_{i\to-\infty} f(i) = -\infty$. For a special case, let substitute $\mathscr{R}_0 = 1$ on $f(i)$, and no positive root of $f(i)$. Let we call this new function as $\bar{f}(i)$. Then we have $i = 0$ as one root of $\bar{f}(i)$. Hence, when $\mathscr{R}_0 > 1$ which means that $b_1 < 0$, then $\bar{f}(i)$ will be shifting downward. Since $\lim_{i\to\infty} f(i) = \infty$, then $f(i)$ will have at least one positive root. In addition, since the expression of s^*, e^*, d^*, l^*, m^* and v^* is always positive when i^* positive, then we have that we always has at least one malaria-endemic equilibrium for $\mathscr{R}_0 > 1$. To illustrate this proof, please see Figure 1. □

Possible existence of malaria-endemic equilibrium when $\mathscr{R}_0 < 1$

Next, we analyze the possibility to have at least one positive roots of $f(i)$ when $\mathscr{R}_0 < 1$. We use gradient analysis on $\mathscr{R}_0 = 1, i = 0$ to understand this possible phenomena. For this purpose, let us define β_1 as a function of $\mathscr{R}_0$ by solving

$$\mathscr{R}_0 = \frac{\xi\,(\kappa_1\mu_h+\eta)\,(\alpha\,\beta_3+\gamma\,\beta_4+\beta_3\mu_h)\,\beta_1}{(\xi+\mu_h)\,(\eta+\mu_h)\,(\mu_h+1)\,(\alpha\,\gamma\,(1-\kappa_2)+\mu_h\alpha+\gamma\,\mu_h+{\mu_h}^2)\,\mu_v} \tag{7}$$

respect to β_1, which give us

$$\beta_1^* = \frac{(\eta+\mu_h)\,(\xi+\mu_h)\,(\mu_h+1)\,\mu_v\left(\alpha\,\gamma\,(1-\kappa_2)+\alpha\,\mu_h+\gamma\,\mu_h+{\mu_h}^2\right)\mathscr{R}_0}{\xi\,(\kappa_1\mu_h+\eta)\,(\alpha\,\beta_3+\gamma\,\beta_4+\beta_3\mu_h)} \tag{8}$$

Substitute β_1^* in (8) in to polynomial $f(i)$ in (6), and take the implicit derivative of i respect to $\mathscr{R}_0$ at $\mathscr{R}_0 = 1, i = 0$ give us:

$$\frac{\partial}{\mathscr{R}_0}\left(c_6(\mathscr{R}_0)\,i^5+c_5(\mathscr{R}_0)\,i^4+c_4(\mathscr{R}_0)\,i^4+c_3(\mathscr{R}_0)\,i^2+c_2(\mathscr{R}_0)\,i+c_1(\mathscr{R}_0)\,\right)=0$$

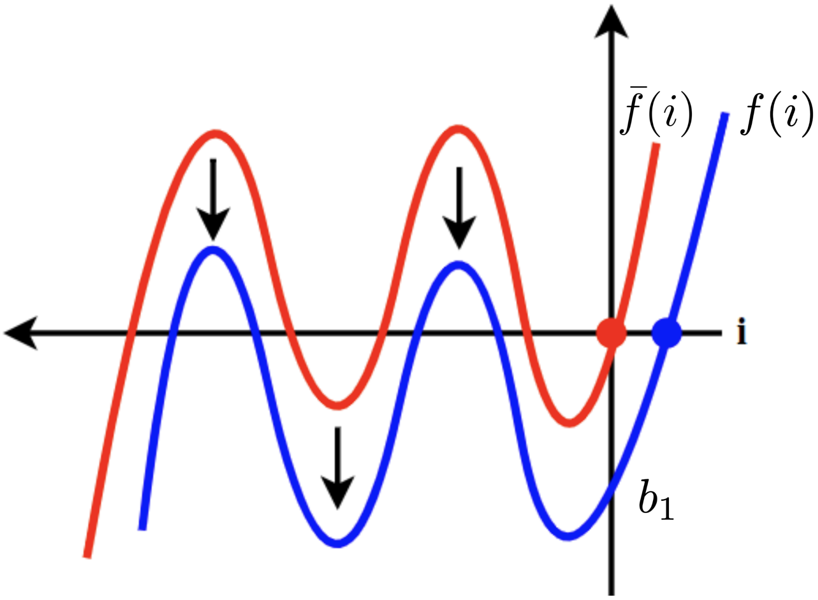

Figure 1: Illustration for the proof of Theorem 2

$$
\begin{aligned}
\iff \quad & \left(\frac{\partial c_6(\mathscr{R}_0)}{\mathscr{R}_0}\, i^5 + 5\, i^4\, c_6\left(\mathscr{R}_0\right) \frac{\partial i}{\partial \mathscr{R}_0}\right) + \left(\frac{\partial c_5(\mathscr{R}_0)}{\mathscr{R}_0}\, i^4 + 4\, i^3\, c_5\left(\mathscr{R}_0\right) \frac{\partial i}{\partial \mathscr{R}_0}\right) \\
& + \left(\frac{\partial c_4(\mathscr{R}_0)}{\mathscr{R}_0}\, i^3 + 3\, i^2\, c_4\left(\mathscr{R}_0\right) \frac{\partial i}{\partial \mathscr{R}_0}\right) + \left(\frac{\partial c_3(\mathscr{R}_0)}{\mathscr{R}_0}\, i^2 + 2\, i\, c_3\left(\mathscr{R}_0\right) \frac{\partial i}{\partial \mathscr{R}_0}\right) \\
& + \left(\frac{\partial c_2(\mathscr{R}_0)}{\mathscr{R}_0}\, i + c_2\left(\mathscr{R}_0\right) \frac{\partial i}{\partial \mathscr{R}_0}\right) + c_1\left(\mathscr{R}_0\right) = 0.
\end{aligned}
$$

Evaluating above equation at the branching point $\mathscr{R}_0 = 1, i = 0$, we have:

$$
c_2\left(\mathscr{R}_0\right) \frac{\partial i}{\partial \mathscr{R}_0} + c_1\left(\mathscr{R}_0\right) = 0.
$$

Solve above equation respect to $\frac{\partial i}{\partial \mathscr{R}_0}$, we get:

$$
\frac{\partial i}{\partial \mathscr{R}_0}|_{i=0,\mathscr{R}_0=1} = \frac{\left(\xi + \mu_h\right)\left(\eta + \mu_h\right)\left(\mu_h + 1\right)\left(\alpha\,\gamma\left(1 - \kappa_2\right) + \mu_h \alpha + \gamma\,\mu_h + {\mu_h}^2\right)\mu_v}{b_{21}\,\beta_1^* + b_{22}} = 0, \tag{9}
$$

where b_{21} and b_{22} has a long expression to be shown in this article. Since the numerator of $\frac{\partial i}{\partial \mathscr{R}_0}$ is always positive, then the sign of $\frac{\partial i}{\partial \mathscr{R}_0}$ is only depend on the sign of the denominator. Solve the denominator of $\frac{\partial i}{\partial \mathscr{R}_0}$ respect to ω, we find that $\frac{\partial i}{\partial \mathscr{R}_0} < 0$ if:

$$
\omega > \omega^* = \frac{\left(\alpha\,\gamma\left(1 - \kappa_2\right) + \alpha\,\mu_h + \gamma\,\mu_h + {\mu_h}^2\right)\left(\alpha\,\beta_3 + \gamma\,\beta_4 + \beta_3\mu_h\right)\, R_1}{\gamma\,\xi\,\mu_v\left(\alpha + \mu_h\right)^2\left(\phi + \mu_h\right)\left(\eta + \mu_h\right)\left(p\mu_h + \eta\right)\left(\alpha\,\beta_3\left(1 - \kappa_2\right) + \left(\beta_3 - \beta_4\right)\mu_h\right)}, \tag{10}
$$

with R_1 is given by:

$$
\begin{aligned}
R_1 \;\; = \;\; & \mu_v(\mu + h + \eta)\left(\mu_h + 1\right)\left(\mu_h + \phi\right)\left[\left(\mu_h + \eta\right)\left(-\alpha\,\gamma\, r + \alpha\,\gamma + \alpha\,\mu_h + \gamma\,\mu_h + {\mu_h}^2\right) \ldots\right. \\
+ \;\; & \left(-\alpha\,\eta\,\gamma\,\phi\, r - \alpha\,\eta\,\gamma\, r\mu_h + \alpha\,\gamma\, p\phi\, r - \alpha\,\gamma\,\phi\, r\mu_h - \alpha\,\gamma\, r{\mu_h}^2 + \alpha\,\eta\,\gamma\,\phi - \alpha\,\eta\,\gamma\, r + \alpha\,\eta\,\gamma\,\mu_h + \alpha\,\eta\,\phi\,\mu_h \ldots\right. \\
+ \;\; & \alpha\,\eta\,{\mu_h}^2 - \alpha\,\gamma\, p\phi - r\alpha\,\gamma\,\phi + \alpha\,\gamma\,\phi\,\mu_h - \alpha\,\gamma\, r\mu_h + \alpha\,\gamma\,{\mu_h}^2 + \alpha\,\phi\,{\mu_h}^2 + \alpha\,{\mu_h}^3 + \eta\,\gamma\,\phi\,\mu_h \ldots \\
+ \;\; & \eta\,\gamma\,{\mu_h}^2 + \eta\,\phi\,{\mu_h}^2 + \eta\,{\mu_h}^3 + \gamma\,\phi\,{\mu_h}^2 + \gamma\,{\mu_h}^3 + \phi\,{\mu_h}^3 + {\mu_h}^4 + \alpha\,\eta\,\gamma + \alpha\,\eta\,\phi + \alpha\,\eta\,\mu_h + \alpha\,\gamma\,\phi \ldots \\
+ \;\; & \left.\left.\alpha\,\gamma\,\mu_h + \alpha\,\phi\,\mu_h + \alpha\,{\mu_h}^2 + \eta\,\gamma\,\phi + \eta\,\gamma\,\mu_h + \eta\,\phi\,\mu_h + \eta\,{\mu_h}^2 + \gamma\,\phi\,\mu_h + \gamma\,{\mu_h}^2 + \phi\,{\mu_h}^2 + {\mu_h}^3\right)\xi\right] \\
+ \;\; & \xi\,\left(\mu_h + \phi\right)\left(\eta\, p\mu_h + p\beta_2\mu_h + p{\mu_h}^2 + \eta^2 + \eta\,\mu_h - \beta_2\mu_h\right)\left(\alpha\,\beta_3 + \gamma\,\beta_4 + \beta_3\mu_h\right).
\end{aligned}
$$

Based on above analysis, we have the following theorem regarding the existence of endemic equilibrium when $\mathscr{R}_0 < 1$.

Theorem 3. *The reduced malaria model in* (4) *is always has at least one malaria-endemic equilibrium point for some interval value on* $\mathscr{R}_0 < 1$ *if* $\omega > \omega^*$.

Possible number of positive equilibrium points

We continue our analysis on the polynomial $f(i)$. Since $f(i)$ is a five-degree polynomial, we have that $f(i)$ can have at most five positive roots. To analyze this possibility, we use the well-known Descartes rule of signs. The result is given in TableS 1.

Table 1: Possible number of positive roots of Polynomial in (6)

Cases	b_6	b_5	b_4	b_3	b_2	b_1	$\mathscr{R}_0$	Number of sign changes	Possible positive roots
1	+	+	+	+	+	+	$\mathscr{R}_0 < 1$	0	0
2	+	+	+	+	+	-	$\mathscr{R}_0 > 1$	1	1
3	+	+	+	+	-	-	$\mathscr{R}_0 > 1$	1	1
4	+	+	+	-	-	-	$\mathscr{R}_0 > 1$	1	1
5	+	+	-	-	-	-	$\mathscr{R}_0 > 1$	1	1
6	+	-	-	-	-	-	$\mathscr{R}_0 > 1$	1	1
7	+	+	+	+	-	+	$\mathscr{R}_0 < 1$	2	0 or 2
8	+	+	+	-	+	+	$\mathscr{R}_0 < 1$	2	0 or 2
9	+	+	+	-	-	+	$\mathscr{R}_0 < 1$	2	0 or 2
10	+	+	-	-	+	+	$\mathscr{R}_0 < 1$	2	0 or 2
11	+	+	-	+	+	+	$\mathscr{R}_0 < 1$	2	0 or 2
12	+	-	-	-	-	+	$\mathscr{R}_0 < 1$	2	0 or 2
13	+	+	-	-	-	+	$\mathscr{R}_0 < 1$	2	0 or 2
14	+	-	+	+	+	+	$\mathscr{R}_0 < 1$	2	0 or 2
15	+	-	-	+	+	+	$\mathscr{R}_0 < 1$	2	0 or 2
16	+	-	-	-	+	+	$\mathscr{R}_0 < 1$	2	0 or 2
17	+	-	-	-	+	-	$\mathscr{R}_0 > 1$	3	1 or 3
18	+	-	-	+	+	-	$\mathscr{R}_0 > 1$	3	1 or 3
19	+	+	-	+	+	-	$\mathscr{R}_0 > 1$	3	1 or 3
20	+	+	+	-	+	-	$\mathscr{R}_0 > 1$	3	1 or 3
21	+	+	-	+	-	-	$\mathscr{R}_0 > 1$	3	1 or 3
22	+	+	-	-	+	-	$\mathscr{R}_0 > 1$	3	1 or 3
23	+	-	+	+	+	-	$\mathscr{R}_0 > 1$	3	1 or 3
24	+	-	+	+	-	-	$\mathscr{R}_0 > 1$	3	1 or 3
25	+	-	+	-	-	-	$\mathscr{R}_0 > 1$	3	1 or 3
26	+	-	-	+	-	-	$\mathscr{R}_0 > 1$	3	1 or 3
27	+	-	+	+	-	+	$\mathscr{R}_0 < 1$	4	0, 2, or 4
28	+	-	+	-	+	+	$\mathscr{R}_0 < 1$	4	0, 2, or 4
29	+	-	+	-	-	+	$\mathscr{R}_0 < 1$	4	0, 2, or 4
30	+	-	-	+	-	+	$\mathscr{R}_0 < 1$	4	0, 2, or 4
30	+	-	-	+	-	+	$\mathscr{R}_0 > 1$	4	0, 2, or 4
32	+	-	+	-	+	-	$\mathscr{R}_0 > 1$	5	1, 3, or 5

Based on TableS 1, we have a conclusions that our malaria model is always had an endemic equilibrium when $\mathscr{R}_0 > 1$, not only one, but it is possible to have 3 or 5 endemic equilibrium when $\mathscr{R}_0 > 1$. Furthermore, we conclude that whenever our model has an endemic equilibrium when $\mathscr{R}_0 < 1$, then it always has 2 or 4 endemic equilibrium. This result indicates a complex dynamic on our proposed model near from the branching point $\mathscr{R}_0 = 1$.

S. 3 Bifurcation analysis

The following simplification and change of variables needed to made the calculation easier to show. Let $x_1 = s, x_2 = e, x_3 = d, x_4 = l, x_5 = i, x_6 = m$, and $x_7 = v$. Hence, if $X = (x_1,\ x_2,\ x_3,\ x_4,\ x_5,\ x_6,\ x_7)^T$, then the malaria model (4) can be rewritten as $\frac{dX}{dt} = F(x)$ with $F = (f_1,\ f_2,\ f_3,\ f_4,\ f_5,\ f_6,\ f_7)^T$.

$$f_1 := \frac{dx_1}{d\tau} = \mu_h - \beta_1\, x_1\, x_7 - \mu_h\, x_1 + \phi\,(1 - x_1 - x_2 - x_3 - x_4 - x_5 - x_6), \tag{11a}$$
$$f_2 := \frac{dx_2}{d\tau} = \beta_1\, x_1\, x_7 - \xi\, x_2 - \mu_h\, x_2, \tag{11b}$$
$$f_3 := \frac{dx_3}{d\tau} = (1 - \kappa_1)\, \xi\, x_2 - \beta_2\, x_3\, x_7 - \eta\, x_3 - \mu_h\, x_3, \tag{11c}$$
$$f_4 := \frac{dx_4}{d\tau} = \kappa_1\, \xi\, x_2 + \beta_2\, x_3\, x_7 + \eta\, x_3 - x_4 - \mu_h\, x_4, \tag{11d}$$
$$f_5 := \frac{dx_5}{d\tau} = \kappa_2\, \alpha\, x_6 - \frac{\gamma\, x_5}{1 + \omega\, x_5} + x_4 - \mu_h\, x_5, \tag{11e}$$
$$f_6 := \frac{dx_6}{d\tau} = \frac{\gamma\, x_5}{1 + \omega\, x_5} - \alpha\, x_6 - \mu_h\, x_6, \tag{11f}$$
$$f_7 := \frac{dx_7}{d\tau} = \beta_3\, x_5\,(1 - x_7) + \beta_4\, x_6\,(1 - x_7) - \mu_v\, x_7. \tag{11g}$$

To use the Castillo-Song bifurcation phenomena, we need to show that system (11) linearized at $\mathscr{R}_0 = 1$ has a simple zero eigenvalue. Let β_1 chosen as the bifurcation parameter, which represent a condition of $\mathscr{R}_0 = 1$. The β_1^* which satisfy $\mathscr{R}_0 = 1$ is given by:

$$\beta_1^* = \frac{(\eta + \mu_h)\,(\xi + \mu_h)\,(\mu_h + 1)\,\mu_v\,\left(\alpha\,\gamma\,(1 - \kappa_2) + \alpha\,\mu_h + \gamma\,\mu_h + {\mu_h}^2\right)\mathscr{R}_0}{\xi\,\left(\kappa_1\mu_h + \eta\right)(\alpha\,\beta_3 + \gamma\,\beta_4 + \beta_3\mu_h)}. \tag{12}$$

Linearized system (11) at β_1^* yield:

$$J(\beta_1^*) = \begin{pmatrix} -\xi - \mu_h & 0 & 0 & 0 & 0 & \beta_1^* \\ (1-\kappa_1)\,\xi & -\eta - \mu_h & 0 & 0 & 0 & 0 \\ \kappa_1\xi & \eta & -\mu_h - 1 & 0 & 0 & 0 \\ 0 & 0 & 1 & -\gamma - \mu_h & \kappa_2\alpha & 0 \\ 0 & 0 & 0 & \gamma & -\alpha - \mu_h & 0 \\ 0 & 0 & 0 & \beta_3 & \beta_4 & -\mu_v \end{pmatrix}. \tag{13}$$

The characteristic polynomial of $J(\beta_1^*)$ is given by:

$$\lambda\,(\lambda + \mu_h + \phi)\,\left(k_5\,\lambda^5 + k_4\,\lambda^4 + k_3\,\lambda^3 + k_2\,\lambda^2 + k_1\,\lambda + k_0\right) = 0,$$

where k_i for $i = 1, 2, \ldots 5$ are positive. Hence, we have a simple zero eigenvalue, and the other eigenvalues are negative. Therefore, we can use the Castillo-Song theorem to proceed to the next step.

Next, we calculate the right eigenvector of $J(\beta_1^*)$ which are denoted as $\mathbf{w} = (w_1,\ w_2,\ w_3,\ w_4,\ w_5,\ w_6,\ w_7)^T$. Solving $J(\beta_1^*)\mathbf{w} = 0$ respect to $\mathbf{w}$, we have

$$\begin{aligned} w_1 &= \frac{w_6}{\gamma\,(\phi + \mu_h)\,(\kappa_1\mu_h + \eta)\,\xi}\,R_3, \\ w_2 &= \frac{(\mu_h + 1)\,(\eta + \mu_h)\,\left(\alpha\,\gamma\,(1 - \kappa_2) + \alpha\,\mu_h + \gamma\,\mu_h + {\mu_h}^2\right)w_6}{\xi\,(\kappa_1\mu_h + \eta)\,\gamma}, \\ w_3 &= \frac{\left(\alpha\,\gamma\,(1 - \kappa_2) + \alpha\,\mu_h + \gamma\,\mu_h + {\mu_h}^2\right)(1 - \kappa_1)\,(\mu_h + 1)\,w_6}{(\kappa_1\mu_h + \eta)\,\gamma}, \\ w_4 &= \frac{\left(\alpha\,\gamma\,(1 - \kappa_2) + \alpha\,\mu_h + \gamma\,\mu_h + {\mu_h}^2\right)w_6}{\gamma}, \\ w_5 &= \frac{(\alpha + \mu_h)\,w_6}{\gamma}, \quad w_6 = w_6, \quad w_7 = \frac{(\alpha\,\beta_3 + \gamma\,\beta_4 + \beta_3\mu_h)\,w_6}{\gamma\,\mu_v}. \end{aligned} \tag{14}$$

Now, we calculate the left eigenvector of $J(\beta_1^*)$ which are denoted as $\mathbf{v} = (v_1,\ v_2,\ v_3,\ v_4,\ v_5,\ v_6,\ v_7)$. Solving $\mathbf{v}J(\beta_1^*) = 0$ respect to $\mathbf{v}$ we have:

$$\begin{aligned}
v_1 &= 0, \quad v_2 = \frac{\xi\ (\kappa_1\mu_h + \eta)\, v_3}{(\xi + \mu_h)\,\eta}, \quad v_3 = v_3, \\
v_4 &= \frac{v_3\,(\eta + \mu_h)}{\eta}, \quad v_5 = \frac{(\mu_h + 1)\,(\eta + \mu_h)\, v_3}{\eta}, \\
v_6 &= \frac{(\eta + \mu_h)\,(\mu_h + 1)\,(\alpha\,\kappa_2\beta_3 + \gamma\,\beta_4 + \beta_4\mu_h)\, v_3}{(\alpha\,\beta_3 + \gamma\,\beta_4 + \beta_3\mu_h)\,\eta}, \\
v_7 &= \frac{(\eta + \mu_h)\,(\mu_h + 1)\left(\alpha\,\gamma\,(1 - \kappa_2) + \alpha\,\mu_h + \gamma\,\mu_h + {\mu_h}^2\right) v_3}{(\alpha\,\beta_3 + \gamma\,\beta_4 + \beta_3\mu_h)\,\eta}.
\end{aligned} \tag{15}$$

With the left and right eigenvector of the zero eigenvalue of $J(\beta_1^*)$ in hand, then we are ready to calculate the coefficients $\mathcal{A}$ and $\mathcal{B}$ of Castillo-Song theorem. By direct calculation, we have

$$\begin{aligned}
\mathcal{A} &= v_2 \sum_{i,j=1}^{6} w_i\, w_j\, \frac{\partial^2 f_2}{\partial x_i \partial x_j} + v_3 \sum_{i,j=1}^{6} w_i\, w_j\, \frac{\partial^2 f_3}{\partial x_i \partial x_j} + v_4 \sum_{i,j=1}^{6} w_i\, w_j\, \frac{\partial^2 f_4}{\partial x_i \partial x_j} \\
&\quad + v_5 \sum_{i,j=1}^{6} w_i\, w_j\, \frac{\partial^2 f_5}{\partial x_i \partial x_j} + v_6 \sum_{i,j=1}^{6} w_i\, w_j\, \frac{\partial^2 f_6}{\partial x_i \partial x_j} + v_7 \sum_{i,j=1}^{6} w_i\, w_j\, \frac{\partial^2 f_7}{\partial x_i \partial x_j} \\
&= v_2\ (2\, w_1\, w_7\, \beta_1) - v_3\ (w_3\, w_7\, \beta_2) + v_4\ (2\, w_3\, w_7\, \beta_2) \\
&\quad + v_5\ (2\, w_5\, w_5\, \gamma\, \omega) - v_6\ (2\, w_5\, w_5\, \gamma\, \omega) - v_7\ (2\, w_5\, w_7\, \beta_3 + 2\, w_6\, w_7\, \beta_4) \\
&= \mathcal{A}_1\, \omega - \mathcal{A}_2,
\end{aligned} \tag{16}$$

where

$$\begin{aligned}
\mathcal{A}_1 &= \gamma\, \xi\, \mu_v\, (\alpha + \mu_h)^2\, (\phi + \mu_h)\, (\eta + \mu_h)\, (p\mu_h + \eta)\, (\alpha\, \beta_3\, (1 - \kappa_2) + (\beta_3 - \beta_4)\, \mu_h)\,, \\
\mathcal{A}_2 &= \left(\alpha\, \gamma\, (1 - \kappa_2) + \alpha\, \mu_h + \gamma\, \mu_h + {\mu_h}^2\right) (\alpha\, \beta_3 + \gamma\, \beta_4 + \beta_3\mu_h)\ R_1,
\end{aligned}$$

where R_1 is given by

$$\begin{aligned}
R_1 &= \mu_v(\mu + h + \eta)\, (\mu_h + 1)\, (\mu_h + \phi)\, \big[(\mu_h + \eta)\, \left(-\alpha\, \gamma\, r + \alpha\, \gamma + \alpha\, \mu_h + \gamma\, \mu_h + {\mu_h}^2\right) \ldots \\
&+ \big(-\alpha\, \eta\, \gamma\, \phi\, r - \alpha\, \eta\, \gamma\, r\mu_h + \alpha\, \gamma\, p\phi\, r - \alpha\, \gamma\, \phi\, r\mu_h - \alpha\, \gamma\, r{\mu_h}^2 + \alpha\, \eta\, \gamma\, \phi - \alpha\, \eta\, \gamma\, r + \alpha\, \eta\, \gamma\, \mu_h + \alpha\, \eta\, \phi\, \mu_h \ldots \\
&+ \alpha\, \eta\, {\mu_h}^2 - \alpha\, \gamma\, p\phi - r\alpha\, \gamma\, \phi + \alpha\, \gamma\, \phi\, \mu_h - \alpha\, \gamma\, r\mu_h + \alpha\, \gamma\, {\mu_h}^2 + \alpha\, \phi\, {\mu_h}^2 + \alpha\, {\mu_h}^3 + \eta\, \gamma\, \phi\, \mu_h \ldots \\
&+ \eta\, \gamma\, {\mu_h}^2 + \eta\, \phi\, {\mu_h}^2 + \eta\, {\mu_h}^3 + \gamma\, \phi\, {\mu_h}^2 + \gamma\, {\mu_h}^3 + \phi\, {\mu_h}^3 + {\mu_h}^4 + \alpha\, \eta\, \gamma + \alpha\, \eta\, \phi + \alpha\, \eta\, \mu_h + \alpha\, \gamma\, \phi \ldots \\
&+ \alpha\, \gamma\, \mu_h + \alpha\, \phi\, \mu_h + \alpha\, {\mu_h}^2 + \eta\, \gamma\, \phi + \eta\, \gamma\, \mu_h + \eta\, \phi\, \mu_h + \eta\, {\mu_h}^2 + \gamma\, \phi\, \mu_h + \gamma\, {\mu_h}^2 + \phi\, {\mu_h}^2 + {\mu_h}^3\big)\, \xi\big] \\
&+ \xi\ (\mu_h + \phi)\, \left(\eta\, p\mu_h + p\beta_2\mu_h + p{\mu_h}^2 + \eta^2 + \eta\, \mu_h - \beta_2\mu_h\right) (\alpha\, \beta_3 + \gamma\, \beta_4 + \beta_3\mu_h)\,.
\end{aligned}$$

We can see from the expression above, that $\mathcal{A} > 0$ if $\omega > \frac{\mathcal{A}_2}{\mathcal{A}_1}$, where $\frac{\mathcal{A}_2}{\mathcal{A}_1}$ is similar with the expression of ω^* (See Supplementary file S2). Next, we calculate $\mathcal{B}$, and find it as follows:

$$\begin{aligned}
\mathcal{B} &= v_2 \sum_{i=1}^{n} w_i\, \frac{\partial^2 f_2}{\partial x_i \partial \beta_1^*} + v_3 \sum_{i=1}^{n} w_i\, \frac{\partial^2 f_3}{\partial x_i \partial \beta_1^*} + v_4 \sum_{i=1}^{n} w_i\, \frac{\partial^2 f_4}{\partial x_i \partial \beta_1^*} \\
&\quad + v_5 \sum_{i=1}^{n} w_i\, \frac{\partial^2 f_5}{\partial x_i \partial \beta_1^*} + v_6 \sum_{i=1}^{n} w_i\, \frac{\partial^2 f_6}{\partial x_i \partial \beta_1^*} + v_7 \sum_{i=1}^{n} w_i\, \frac{\partial^2 f_7}{\partial x_i \partial \beta_1^*} \\
&= \frac{\xi\ (\kappa_1\mu_h + \eta)\, v_3\, (\alpha\, \beta_3 + \gamma\, \beta_4 + \beta_3\mu_h)\, w_6}{(\xi + \mu_h)\, \eta\gamma\, \mu_v}.
\end{aligned} \tag{17}$$

From the expression of $\mathcal{B}$, we can see that $\mathcal{B}$ is always positive for all positive parameters in system (11). According to these results, we have the following theorem.

Theorem 4. *The malaria model* (4) *undergoes a backward bifurcation phenomena at* $\mathcal{R}_0 = 1$ *if* $\omega > \omega^*$, *where*

$$\omega > \omega^* = \frac{\left(\alpha\, \gamma\, (1 - \kappa_2) + \alpha\, \mu_h + \gamma\, \mu_h + {\mu_h}^2\right) (\alpha\, \beta_3 + \gamma\, \beta_4 + \beta_3\mu_h)\ R_1}{\gamma\, \xi\, \mu_v\, (\alpha + \mu_h)^2\, (\phi + \mu_h)\, (\eta + \mu_h)\, (p\mu_h + \eta)\, (\alpha\, \beta_3\, (1 - \kappa_2) + (\beta_3 - \beta_4)\, \mu_h)}. \tag{18}$$

S. 4 Characterization of the optimal control problem

The optimal control model read as:

$$
\begin{aligned}
\frac{dS}{dt} &= \Lambda_h - (1-u_1(t)\zeta)\frac{\bar{\beta}_1 SV}{N} - \bar{\mu}_h S + \bar{\phi}\,R,\\
\frac{dE}{dt} &= (1-u_1(t)\zeta)\frac{\bar{\beta}_1 SV}{N} - \bar{\xi}\,E - \bar{\mu}_h E,\\
\frac{dD}{dt} &= (1-\kappa_1)\,\bar{\xi}\,E - (1-u_1(t)\zeta)\frac{\bar{\beta}_2 DV}{N} - \bar{\eta}\,D - \bar{\mu}_h D,\\
\frac{dL}{dt} &= \kappa_1\bar{\xi}\,E + (1-u_1(t)\zeta)\frac{\bar{\beta}_2 DV}{N} + \bar{\eta}\,D - \bar{\epsilon}\,L - \bar{\mu}_h L,\\
\frac{dI}{dt} &= \kappa_2\,\bar{\alpha}\,T + \bar{\epsilon}\,L - \frac{u_2(t)\,I}{1+\bar{\omega}\,I} - \bar{\mu}_h I,\\
\frac{dT}{dt} &= \frac{u_2(t)\,I}{1+\bar{\omega}\,I} - \bar{\alpha}\,T - \bar{\mu}_h T,\\
\frac{dR}{dt} &= (1-\kappa_2)\;\bar{\alpha}\,T - \bar{\phi}\,R - \bar{\mu}_h R,\\
\frac{dU}{dt} &= \Lambda_v - (1-u_1(t)\zeta)\frac{\bar{\beta}_3 UI}{N} - (1-u_1(t)\zeta)\frac{\bar{\beta}_4\,U\,T}{N} - (\bar{\mu}_v + u_3(t))U,\\
\frac{dV}{dt} &= (1-u_1(t)\zeta)\frac{\bar{\beta}_3 UI}{N} + (1-u_1(t)\zeta)\frac{\bar{\beta}_4\,U\,T}{N} - (\bar{\mu}_v + u_3(t))V.
\end{aligned} \tag{19}
$$

We define the Hamiltonian function by applying the Pontryagin's Maximum Principle as follows:

$$
\begin{aligned}
\mathcal{H}(t,\boldsymbol{x},\boldsymbol{u},\lambda) = {}& \omega_1 E + \omega_2 D + \omega_3 L + \omega_4 I + \varphi_1 u_1^2 + \varphi_2 u_2^2 + \varphi_3 u_3^2\\
+ {}& \left(\Lambda_h - (1-u_1(t)\zeta)\frac{\overline{\beta}_1 SV}{N} - \overline{\mu}_h S + \overline{\phi} R\right)\lambda_1\\
+ {}& \left((1-u_1(t)\zeta)\frac{\overline{\beta}_1 SV}{N} - \overline{\xi} E - \overline{\mu}_h E\right)\lambda_2\\
+ {}& \left((1-\kappa_1)\,\overline{\xi} E - (1-u_1(t)\zeta)\frac{\overline{\beta}_2 DV}{N} - \overline{\eta} D - \overline{\mu}_h D\right)\lambda_3\\
+ {}& \left(\kappa_1 \overline{\xi} E + (1-u_1(t)\zeta)\frac{\overline{\beta}_2 DV}{N} + \overline{\eta} D - \overline{\epsilon} L - \overline{\mu}_h L\right)\lambda_4\\
+ {}& \left(\kappa_2 \overline{\alpha} T + \overline{\epsilon} L - \frac{u_2(t)\,I}{1+\overline{\omega} I} - \overline{\mu}_h I\right)\lambda_5\\
+ {}& \left(\frac{u_2(t)\,I}{1+\overline{\omega} I} - \overline{\alpha} T - \overline{\mu}_h T\right)\lambda_6\\
+ {}& \left((1-\kappa_2)\,\overline{\alpha} T - \overline{\phi} R - \overline{\mu}_h R\right)\lambda_7\\
+ {}& \left(\Lambda_v - (1-u_1(t)\zeta)\frac{\overline{\beta}_3 UI}{N} - (1-u_1(t)\zeta)\frac{\overline{\beta}_4 UT}{N} - (\overline{\mu}_v + u_3(t))\,U\right)\lambda_8,\\
+ {}& \left((1-u_1(t)\zeta)\frac{\overline{\beta}_3 UI}{N} + (1-u_1(t)\zeta)\frac{\overline{\beta}_4 UT}{N} - (\overline{\mu}_v + u_3(t))\,V\right)\lambda_9.
\end{aligned}
$$

Thus, taking the partial derivatives of $\mathcal{H}$ with respective to each state variables yields the given adjoint system below:

$$
\begin{aligned}
\frac{d\lambda_1}{dt} = -\frac{d\mathcal{H}}{dS} &= \left(\frac{(1-u_1(t)\zeta)\overline{\beta}_1 VN - (1-u_1(t)\zeta)\overline{\beta}_1 SV}{N^2}\right)(\lambda_1-\lambda_2) \\
&+ \left(\frac{(1-u_1(t)\zeta)\overline{\beta}_2 DV}{N^2}\right)(\lambda_4-\lambda_3) + \left(\frac{(1-u_1(t)\zeta)\overline{\beta}_3 UI + (1-u_1(t)\zeta)\overline{\beta}_4 UT}{N^2}\right)(\lambda_9-\lambda_8) + \mu_h\lambda_1 \\
\frac{d\lambda_2}{dt} = -\frac{d\mathcal{H}}{dE} &= -\omega_1 + \left(\frac{(1-u_1(t)\zeta)\overline{\beta}_1 SV}{N^2}\right)(\lambda_2-\lambda_1) + \left(\frac{(1-u_1(t)\zeta)\overline{\beta}_2 DV}{N^2}\right)(\lambda_4-\lambda_3) \\
&+ \left(\frac{(1-u_1(t)\zeta)\overline{\beta}_3 UI + (1-u_1(t)\zeta)\overline{\beta}_4 UT}{N^2}\right)(\lambda_9-\lambda_8) + \xi(\lambda_2-\lambda_3) + \kappa_1\xi(\lambda_3-\lambda_4) + \mu_h\lambda_2, \\
\frac{d\lambda_3}{dt} = -\frac{d\mathcal{H}}{dD} &= -\omega_2 + \left(\frac{(1-u_1(t)\zeta)\overline{\beta}_1 SV}{N^2}\right)(\lambda_2-\lambda_1) \\
&+ \left(\frac{(1-u_1(t)\zeta)\overline{\beta}_2 VN - (1-u_1(t)\zeta)\overline{\beta}_2 DV}{N^2}\right)(\lambda_3-\lambda_4) \\
&+ \left(\frac{(1-u_1(t)\zeta)\overline{\beta}_3 UI + (1-u_1(t)\zeta)\overline{\beta}_4 UT}{N^2}\right)(\lambda_9-\lambda_8) + \eta(\lambda_3-\lambda_4) + \mu_h\lambda_3, \\
\frac{d\lambda_4}{dt} = -\frac{d\mathcal{H}}{dL} &= -\omega_3 + \left(\frac{(1-u_1(t)\zeta)\overline{\beta}_1 SV}{N^2}\right)(\lambda_2-\lambda_1) + \left(\frac{(1-u_1(t)\zeta)\overline{\beta}_2 DV}{N^2}\right)(\lambda_4-\lambda_3) \\
&+ \left(\frac{(1-u_1(t)\zeta)\overline{\beta}_3 UI + (1-u_1(t)\zeta)\overline{\beta}_4 UT}{N^2}\right)(\lambda_9-\lambda_8) + \epsilon(\lambda_4-\lambda_5) + \mu_h\lambda_4, \\
\frac{d\lambda_5}{dt} = -\frac{d\mathcal{H}}{dI} &= -\omega_4 + \left(\frac{(1-u_1(t)\zeta)\overline{\beta}_1 SV}{N^2}\right)(\lambda_2-\lambda_1) + \left(\frac{(1-u_1(t)\zeta)\overline{\beta}_2 DV}{N^2}\right)(\lambda_4-\lambda_3) \\
&+ \left(\frac{(1-u_1(t)\zeta)\overline{\beta}_3 UN - (1-u_1(t)\zeta)\overline{\beta}_3 UI}{N^2}\right)(\lambda_8-\lambda_9) + \left(\frac{(1-u_1(t)\zeta)\overline{\beta}_4 UT}{N^2}\right)(\lambda_9-\lambda_8) + \mu_h\lambda_5 \\
&+ \left(\frac{u_2(t)}{(1+\varpi I)^2}\right)(\lambda_5-\lambda_6), \\
\frac{d\lambda_6}{dt} = -\frac{d\mathcal{H}}{dT} &= \left(\frac{(1-u_1(t)\zeta)\overline{\beta}_1 SV}{N^2}\right)(\lambda_2-\lambda_1) + \left(\frac{(1-u_1(t)\zeta)\overline{\beta}_2 DV}{N^2}\right)(\lambda_4-\lambda_3) \\
&+ \left(\frac{(1-u_1(t)\zeta)\overline{\beta}_4 UN - (1-u_1(t)\zeta)\overline{\beta}_4 UT}{N^2}\right)(\lambda_8-\lambda_9) + \alpha(\lambda_6-\lambda_7) + \kappa_2\alpha(\lambda_7-\lambda_5) + \mu_h\lambda_6 \\
&+ \left(\frac{(1-u_1(t)\zeta)\overline{\beta}_3 UI}{N^2}\right)(\lambda_9-\lambda_8), \\
\frac{d\lambda_7}{dt} = -\frac{d\mathcal{H}}{dR} &= \left(\frac{(1-u_1(t)\zeta)\overline{\beta}_1 SV}{N^2}\right)(\lambda_2-\lambda_1) + \left(\frac{(1-u_1(t)\zeta)\overline{\beta}_2 DV}{N^2}\right)(\lambda_4-\lambda_3) \\
&+ \left(\frac{(1-u_1(t)\zeta)\overline{\beta}_3 UI + (1-u_1(t)\zeta)\overline{\beta}_4 UT}{N^2}\right)(\lambda_9-\lambda_8) + \phi(\lambda_7-\lambda_1) + \mu_h\lambda_7, \\
\frac{d\lambda_8}{dt} = -\frac{d\mathcal{H}}{dU} &= \left(\frac{(1-u_1(t)\zeta)\overline{\beta}_3 IN + (1-u_1(t)\zeta)\overline{\beta}_4 TN}{N^2}\right)(\lambda_8-\lambda_9) + (\mu_v + u_3(t))\lambda_8, \\
\frac{d\lambda_9}{dt} = -\frac{d\mathcal{H}}{dV} &= \left(\frac{(1-u_1(t)\zeta)\overline{\beta}_1 SN}{N^2}\right)(\lambda_1-\lambda_2) + \left(\frac{(1-u_1(t)\zeta)\overline{\beta}_2 DN}{N^2}\right)(\lambda_3-\lambda_4) + (\mu_v + u_3(t))\lambda_9.
\end{aligned}
$$

Here, it is of great importance to note that ((20)) were obtained by considering the completed transversality conditions for which $\lambda_j(t) = 0$, in which $j = 1, 2, \ldots, 9$. Next, we solve for the control variables u_i for $i = 1, 2, 3$ for their respective optimality conditions by solving $\frac{d\mathcal{H}}{du_i} = 0$, which yields:

$$
\begin{aligned}
u_1^* &= \frac{\beta_1 SV\zeta}{2\varphi_1 N}(\lambda_2-\lambda_1) + \frac{\beta_2 DV\zeta}{2\varphi_1 N}(\lambda_4-\lambda_3) + \left(\frac{\beta_3 UI\zeta + \beta_2 UT\zeta}{2\varphi_1 N}\right)(\lambda_9-\lambda_8) \\
u_2^* &= \frac{I}{2\varphi_2(1+\omega I)}(\lambda_5-\lambda_6) \\
u_3^* &= \frac{U\lambda_8 + V\lambda_9}{2\varphi_3}.
\end{aligned} \tag{20}
$$

Now, using the upper and lower constraints on the admissible controls we have the optimal intervention as given by

$$
\begin{aligned}
u_1^* &= \min\left\{1, \max\left(0, \frac{\beta_1 SV\zeta}{2\varphi_1 N}(\lambda_2-\lambda_1) + \frac{\beta_2 DV\zeta}{2\varphi_1 N}(\lambda_4-\lambda_3) + \left(\frac{\beta_3 UI\zeta + \beta_2 UT\zeta}{2\varphi_1 N}\right)(\lambda_9-\lambda_8)\right)\right\}, \\
u_2^* &= \min\left\{1, \max\left(0, \frac{I}{2\varphi_2(1+\omega I)}(\lambda_5-\lambda_6)\right)\right\}, \\
u_3^* &= \min\left\{1, \max\left(0, \frac{U\lambda_8 + V\lambda_9}{2\varphi_3}\right)\right\}.
\end{aligned} \tag{21}
$$
